# Genome-wide identification and analysis of *B-BOX* gene family in grapevine reveal its potential functions in berry development

**DOI:** 10.1186/s12870-020-2239-3

**Published:** 2020-02-13

**Authors:** Hongru Wei, Peipei Wang, Jianqing Chen, Changjun Li, Yongzhang Wang, Yongbing Yuan, Jinggui Fang, Xiangpeng Leng

**Affiliations:** 1 Qingdao Key Lab of Modern Agriculture Quality and Safety Engineering, College of Horticulture, Qingdao Agricultural University, Qingdao, 266109 People’s Republic of China; 2 College of Horticulture, Nanjing Agricultural University, Nanjing, 210095 China; 3 College of Horticulture, Fujian Agriculture and Forestry University, Fuzhou, 350002 China; 4 Institute of Grape Science and Engineering, College of Horticulture, Qingdao Agricultural University, Qingdao, 266109 People’s Republic of China

**Keywords:** B-BOX, Grapevine, Gene expression, Berry development, Stress response

## Abstract

**Background:**

The B-BOX (BBX) proteins are the class of zinc-finger transcription factors and can regulate plant growth, development, and endure stress response. In plants, the BBX gene family has been identified in *Arabidopsis*, rice, and tomato. However, no systematic analysis of BBX genes has been undertaken in grapevine.

**Results:**

In this study, 24 grapevine *BBX* (*VvBBX*) genes were identified by comprehensive bioinformatics analysis. Subsequently, the chromosomal localizations, gene structure, conserved domains, phylogenetic relationship, gene duplication, and *cis*-acting elements were analyzed. Phylogenetic analysis divided *VvBBX* genes into five subgroups. Numerous *cis*-acting elements related to plant development, hormone and/or stress responses were identified in the promoter of the *VvBBX* genes. The tissue-specific expressional dynamics of *VvBBX* genes demonstrated that *VvBBXs* might play important role in plant growth and development. The transcript analysis from transcriptome data and qRT-PCR inferred that 11 *VvBBX* genes were down-regulated in different fruit developmental stages, while three *VvBBX* genes were up-regulated. It is also speculated that *VvBBX* genes might be involved in multiple hormone signaling (ABA, ethylene, GA3, and CPPU) as transcriptional regulators to modulate berry development and ripening. *VvBBX22* seems to be responsive to multiple hormone signaling, including ABA, ethylene GA3, and CPPU. Some *VvBBX* genes were strongly induced by Cu, salt, waterlogging, and drought stress treatment. Furthermore, the expression of *VvBBX22* proposed its involvement in multiple functions, including leaf senescence, abiotic stress responses, fruit development, and hormone response.

**Conclusions:**

Our results will provide the reference for functional studies of *BBX* gene family, and highlight its functions in grapevine berry development and ripening. The results will help us to better understand the complexity of the *BBX* gene family in abiotic stress tolerance and provide valuable information for future functional characterization of specific genes in grapevine.

## Background

Zinc-finger transcription factor is one of the most important family in the plant kingdom, which play essential role in plant growth, development, and response to environmental stimuli [1, 2]. Zinc finger transcription factors are divided further into several subfamilies according to the structural and functional features of their individual members. The BBX proteins belong to zinc-finger transcription factors and attracted more attention in recent years due to their multiple functions. BBX proteins in plants are comprised of one or two conserved BBX domains in the N-terminus and occasionally a CCT (CONSTANS, CO-like, and TOC1) domain in the C-terminus. B-BOX motifs play significant role in protein-protein interactions and transcriptional regulation [3, 4]. The CCT domain participates in nuclear transport and transcriptional regulation [5–7]. In *Arabidopsis*, 32 BBX proteins have been identified and these members are classified into five subgroups depending on the presence of B-BOX domains along with the CCT domain [3]. Subsequently, increasing evidence suggests that plant BBX proteins play pivotal role in diverse physiological and biochemical processes, such as flower induction [8, 9], photomorphogenesis [10, 11], shade avoidance response [12], carotenoid biosynthesis [13], and biotic and abiotic stress response [14, 15].

*CONSTANS* (*CO*)*/AtBBX1* is the first investigated *BBX* gene in *Arabidopsis*, which is a central coordinator in controlling flowering time by triggering the expression of *Flowering Locus T* (*FT*) gene [16–18]. *co* mutants delay flowering significantly under a long-day, whereas co-overexpressed transgenic plants flower early in both long and short-day conditions [19–21]. There are other *BBX* genes, such as *BBX4*, *BBX7,* and *BBX32*, which have been found to regulate the flowering times [22–24]. At least ten BBX genes have been identified as a regulators of the early photomorphogenesis. Out of ten, *BBX4*, *BBX21*, *BBX22*, and *BBX23* positively regulate plant photomorphogenesis [22, 25–27], whereas *BBX19*, *BBX20*, *BBX24*, *BBX25*, *BBX28* and *BBX32* are negative regulator of photomorphogenesis in *Arabidopsis* [11, 28–30]. For example, *BBX28* negatively regulates photomorphogenesis by repressing the expression of *HY5* and undergoes COP1-mediated degradation [11]. Several *BBX* genes also show their functions in shade avoidance by mediating cell elongation [12, 31, 32].

The BBX proteins are also involved in abiotic stress response and hormonal signaling networks. For example, *BBX18* and *BBX23* are positive thermomorphogenesis regulators and the deficient mutations of *BBX18* and *BBX23* result in reduced thermoresponsive hypocotyl elongation [15]. In *Arabidopsis*, BBX24 is initially isolated as a salt tolerant protein (STO) and increases salt tolerance activities in yeast cells [33]. Overexpression of *BBX24* in *Arabidopsis* enhances the growth of roots under high salinity conditions [34]. In *Chrysanthemum*, *CmBBX24* also enhances cold and drought tolerance besides delaying flowering time [18]. *BBX* genes also reveal their functions in phytohormone signal transduction. *AtBBX18* (*AtDBB1a*) is a positive regulator in the gibberellin (GA) signaling pathway [35], whereas *BBX20* (*AtBZS1*) negatively regulates the brassinosteroid signaling network [28]. Interestingly, BBX proteins also show their importance in the development of fruits, especially in anthocyanin and carotenoid biosynthesis. *MdBBX22* (*MdCOL11*) is involved in MdHY5-mediated signal transduction and modulates anthocyanin accumulation in apple peel [36]. *SlBBX20* promotes chloroplast development and carotenoid accumulation in tomato by directly activating the expression PHYTOENE SYNTHASE 1 [13].

Grapevine is one of the most widely cultivated and commonly consumed fruit crops throughout the world due to its economic importance and essential nutrition [37, 38]. Although the BBX family has been identified in *Arabidopsis*, tomato, pear, and apple [3, 39–41], yet no comprehensive study of *BBX* genes in grapevine has been reported so far. With the release of the grapevine genome [42], we have a better possibility to systematically investigate the putative functions of *BBX* genes in grapevine. In this study, 24 non-redundant members of the *VvBBX* gene family were characterized in grapevine. Subsequently, the detailed gene structures, phylogenetic relationships, tissue expression profiles and expression profiles under different stress conditions were investigated. Our results of *VvBBX* genes will provide a foundation for further functional characterization of *BBX* genes in grapevine.

## Results

### Identification of *VvBBX* genes in grapevine

To identify and obtain the BBX genes in the grapevine genome, the *Arabidopsis* BBX proteins were used as a query to search against the local grapevine genome database by using DNAtools software. Then, the hidden Markov model (HMM) profile of the B-box domain (Pfam00643) was employed to perform a global search of the grapevine genome. After analyzing the conserved domain and removing the redundant sequences, a total of 24 putative *VvBBX* genes were identified in grapevine. For the sake of nomenclature and consistency, these *VvBBX* genes were named from *VvBBX1* to *VvBBX24* depending on their homology to the pear *BBX* members, based on their similar numbers of *BBX* family between grapevine and pear [40]. The detailed information of VvBBX was listed in Table 1, including gene name, protein length, chromosome location, molecular weight, theoretical isoelectric point, aliphatic index, and GRAVY. The 24 VvBBX proteins had diverse molecular length and weight, ranging from 127 (VvBBX22) to 474 (VvBBX7) in amino acid length. VvBBX22 showed the lowest value of the molecular weight (14.26 kDa), while the highest of the molecular weight (51.43 kDa) was observed in VvBBX7. Theoretical isoelectric points of these VvBBX proteins varied from 4.29 (VvBBX21) to 9.01 (VvBBX24) and the value of the aliphatic index ranged from 47.66 (VvBBX23) to 78.38 (VvBBX24). The GRAVY of all VvBBXs was less than zero, indicating the hydrophilic nature of VvBBX proteins (Table 1). The majority of VvBBX proteins were predicted to be located on the nucleus by WoLF PSORT, but a few of them may be located in other subcellular compartments, such as chloroplast and cytoplasm (Table 1).

**Table 1:** BBX gene family in grapevine

Gene Name	Accession number	Protein/AA	Chrom	Chr srart	Chr end	MW (Da)	pI	Aliphatic index	GRAVY	Loc
VvBBX1	VIT_214s0083g00640.2	391	Chr14	22,696,209	22,698,176	42,699.57	5.59	63.94	−0.533	Nucl:6, Chlo:5, Cyto:2
VvBBX2	VIT_211s0052g01800.1	361	Chr11	19,620,989	19,622,158	38,993.62	6.31	60.89	−0.415	Chlo:8, Cyto:2, mito: 2
VvBBX3	VIT_204s0008g07340.1	347	Chr4	7,669,873	7,671,263	38,001.35	5.93	65.82	−0.486	Chlo:14
VvBBX4	VIT_212s0057g01350.2	465	Chr12	10,078,174	10,083,268	51,070.96	5.20	63.14	−0.418	Nucl:12, Cyto:1
VvBBX5	VIT_200s0194g00070.1	414	ChrUn	10,576,010	10,585,823	45,041.34	5.29	69.98	−0.401	Nucl:13
VvBBX6	VIT_207s0104g01360.1	394	Chr7	2,380,513	2,383,530	43,918.32	5.84	70.53	−0.533	Nucl:9, Chlo:2, Cyto:2
VvBBX7	VIT_201s0146g00360.1	474	Chr1	22,216,468	22,220,026	51,428.42	5.89	67.07	−0.523	Nucl:9, Cyto:2, Chlo:1
VvBBX8	VIT_214s0068g01380.1	449	Chr14	25,084,927	25,088,322	49,441.06	5.79	63.61	−0.607	Nucl:5, Cyto:5, Chlo:2
VvBBX9	VIT_201s0011g04240.1	357	Chr1	3,849,985	3,854,088	39,946.58	4.97	74.51	−0.447	Nucl:9, Cyto:2, Extr:2
VvBBX10	VIT_219s0014g05120.1	375	Chr19	5,407,427	5,409,093	41,882.27	5.28	67.81	−0.367	Nucl:13
VvBBX11	VIT_212s0059g02500.1	434	Chr12	7,291,808	7,293,953	47,040.03	7.68	64.54	−0.381	Nucl:10, Chlo:2, Cyto:1
VvBBX12	VIT_201s0011g03520.1	452	Chr1	3,190,707	3,192,646	50,080.42	5.47	65.91	−0.535	Chlo:5, Cyto:5, Nucl:3
VvBBX13	VIT_203s0038g00690.1	210	Chr3	596,303	598,436	23,371.44	5.99	65.95	−0.596	Cyto:7, Nucl:3, Chlo:2
VvBBX14	VIT_204s0023g03030.1	197	Chr4	19,619,418	19,622,085	21,766.85	6.37	73.30	−0.429	Cyto:5, Chlo:3, Nucl:3
VvBBX15	VIT_218s0001g13520.1	303	Chr18	11,546,682	11,547,810	33,157.15	6.19	69.54	−0.444	Nucl:9, Cyto:3, Chlo:1
VvBBX16	VIT_203s0038g00340.1	302	Chr3	310,992	312,061	33,371.52	7.96	64.64	−0.551	Nucl:13
VvBBX17	VIT_218s0089g01280.1	205	Chr18	29,194,471	29,196,480	22,812.56	5.20	64.63	−0.439	Nucl:14
VvBBX18	VIT_219s0014g00350.1	293	Chr19	357,072	371,413	31,682.56	5.07	64.61	−0.332	Chlo:7, Nucl:6
VvBBX19	VIT_205s0102g00750.1	239	Chr5	22,644,172	22,646,527	26,443.94	4.86	71.97	−0.387	Nucl:12, Chlo:1
VvBBX20	VIT_219s0014g03960.1	299	Chr19	4,194,626	4,195,896	32,867.54	4.46	57.16	−0.922	Nucl:10, Mito: 2, Chlo:1
VvBBX21	VIT_212s0134g00400.1	299	Chr12	8,048,666	8,049,880	32,306.61	4.29	53.58	−0.855	Nucl:11, Chlo:1, Cyto:1
VvBBX22	VIT_212s0059g02510.1	127	Chr12	7,296,673	7,297,056	14,258.41	8.02	76.06	−0.202	Nucl:10, Chlo:2, Cyto:1
VvBBX23	VIT_200s0203g00210.1	188	ChrUn	12,064,339	12,064,986	20,392.09	4.46	47.66	−0.849	Nucl:8, Chlo:3, Extr:2
VvBBX24	VIT_209s0054g00530.1	260	Chr9	21,166,877	21,167,659	28,155.08	9.01	78.38	−0.216	Chlo:6, Nucl: 2, Plas:2

*AA* amino acid residues, *Chrom* chromosome, *MW* molecular weight, *pI* theoretical isoelectric point, *GRAVY*, grand average of hydropathicity, *Loc* subcellular location. The subcellular location results of grapevine BBX genes were predicted by WoLF PSORT (https://www.genscript.com/wolf-psort.html). *Nucl* nucleus, *Chlo* chloroplast, *Cyto* cytosol, *Mito* mitochondria, *Plas* plasma membrane, *Extr* extracellular. Testk used for kNN is: 14

### Protein sequence and phylogenetic analysis of the VvBBX gene family

The length of VvBBX proteins varied widely from 127 to 474 amino acids. Among them, eight VvBBXs were found to contain two B-BOX domains and a conserved CCT domain. Nine members consisted of two B-BOX domains but no CCT domain. Two VvBBX contained one B-BOX plus a CCT domain, and five with only one B-BOX domain (Additional file 1: Figure S1). The protein sequence alignment and motifs logos showed that the B-Box 1 and B-Box 2 domains of the VvBBXs had similar conserved amino acid residues, and the CCT domain was also highly conserved among the VvBBX proteins (Additional file 2: Figure S2 and Additional file 3: Figure S3). The motifs logos of these domains were shown in Additional file 2: Figure S2, and their correspondence locations were illustrated in Additional file 3: Figure S3.

**Fig. 1:**
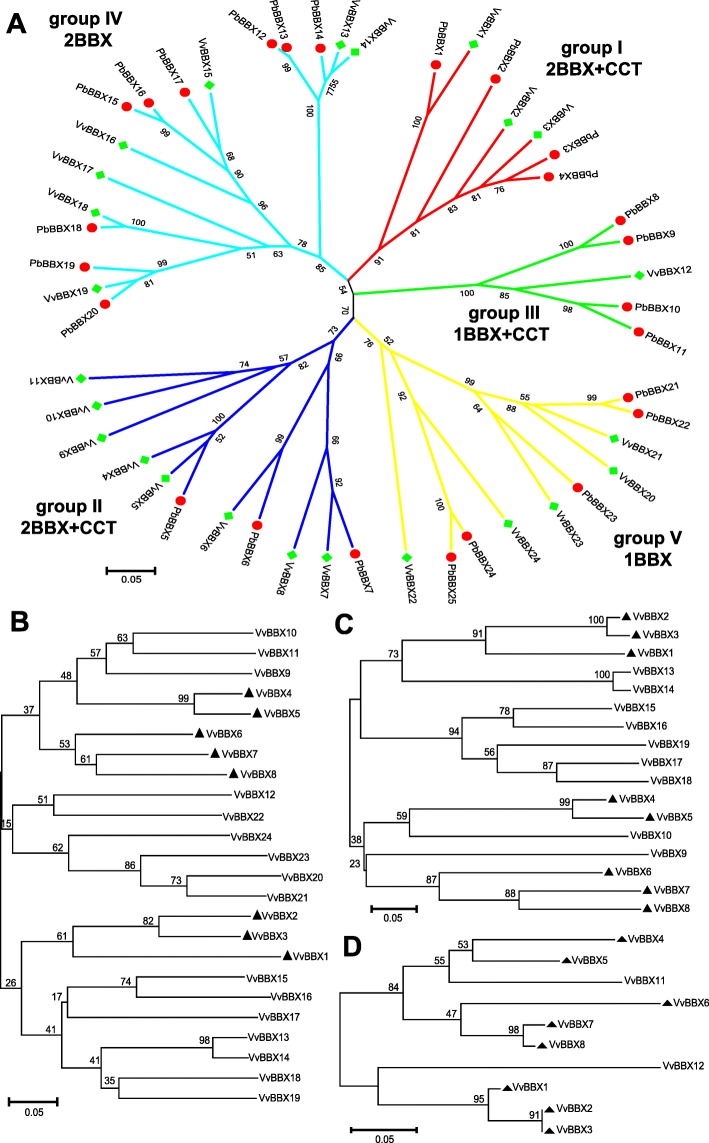
Phylogenetic analysis of BBX genes in grapevine and pear. **a** The full-length amino acid sequences of BBX proteins from grapevine (VvBBX) and pear (PbBBX) were aligned by ClustalX, and the phylogenetic tree was constructed using the neighbor-joining method with1000 bootstrap replicates by MEGA5.0. **b**, **c**, **d** The trees shown were based on the alignments of the protein sequences of the B-Box 1 domain, B-Box 2 domain and CCT domain, respectively. The bootstrap values were indicated at each node. The members marked in black triangle contain two B-BOX and one CCT domains

To explore the evolutionary relationship and functional divergence of the VvBBX members, full-length amino acid sequences of VvBBXs and pear BBXs (PbBBXs) was used to construct the phylogenetic tree by using the neighbor-joining and maximum likelihood method in MEGA, respectively (Fig. 1a and Additional file 4: Figure S4). As shown in the phylogenetic tree (Fig. 1a and Additional file 4: Figure S4), the VvBBX family was divided into five subgroups according to the phylogenetic analysis and consistent with the previous study in pear, tomato, and *Arabidopsis* [3, 39, 40]. Additionally, to gain a better understanding of the classification of BBX members, the phylogenetic tree of VvBBXs, together with PbBBXs, AtBBXs, and SlBBXs was also constructed by using the maximum likelihood method (Additional file 5: Figure S5). As shown in the phylogenetic tree (Additional file 5: Figure S5), all BBX proteins were also divided into five subgroups and most BBX members from grapevine and pear clustered together. Furthermore, the sequences of B-Box 1 (Fig. 1b), B-Box 2 (Fig. 1c) and CCT (Fig. 1d) domain were also employed for the phylogenetic analysis, respectively. The members of subgroups I, II and III contained both B-BOX and CCT domains except for VvBBX9 and VvBBX10, which only owned two B-BOX domains. On the contrary, the members in subgroup IV and V had no CCT domain and only contained two or one B-BOX domain(s), respectively. Eight VvBBXs in subgroup I and II contained all the three domains, and they could be divided into two different groups (Fig. 1b, c, and d). Three of them (VvBBX1, 2, 3) were clustered together in subgroup I, while the other five members from subgroup II also aligned together.

### Chromosomal localization, gene duplication and gene structure analysis of VvBBXs

**Fig. 2:**
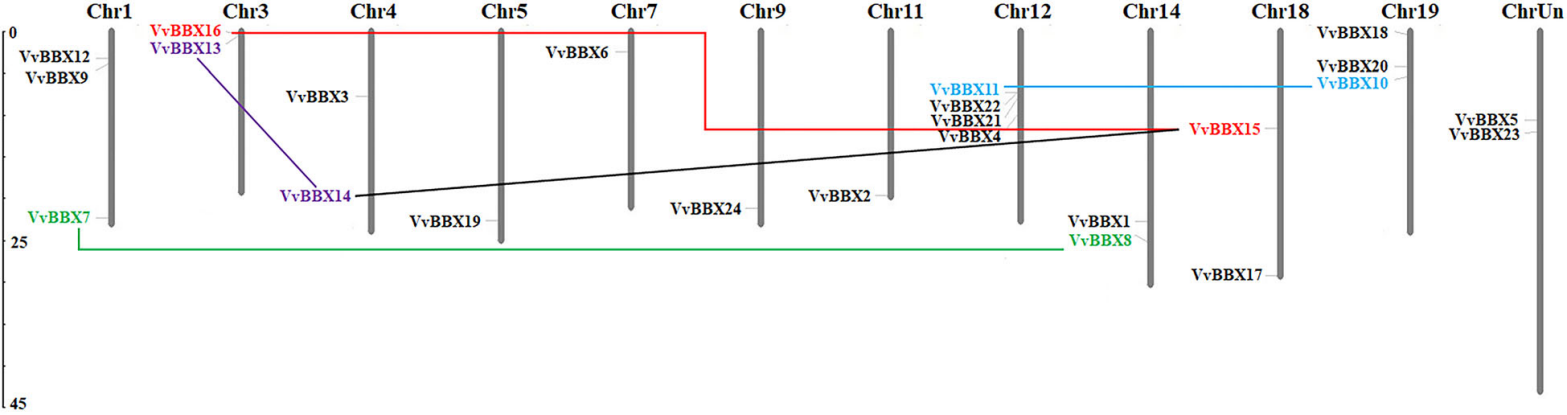
Chromosome distribution and segmental duplication of grapevine BBX genes. Chromosomal mapping was based on the physical position (Mb) in 12 grapevine chromosomes. The scale on the left is in megabases (Mb). The chromosome numbers are indicated at the top of each bar. Five pairs of the segmental duplicated genes are indicated in a different color and are connected by lines

To understand the genomic distribution and gene duplication of *VvBBX* genes, 22 *VvBBX* genes were distributed unevenly throughout the 11 out of the 19 chromosomes and the remaining two genes (*VvBBX5* and *VvBBX23*) had not yet been assembled to any chromosome according to the current grapevine genome (Fig. 2). Among them, the chromosomes 12 had the highest number of *VvBBX* genes (four), while only one *VvBBX* gene was localized on chromosome 5, 7, 9 and 11. Three *VvBBX* genes were located on each of chromosomes 1 and 19 and two *VvBBX* genes were distributed on chromosome 3, 4, 14 and 18, respectively (Fig. 2).

Tandem duplication and segmental duplications occurred frequently in gene families evolution and expansion. Tandem duplication usually caused gene clusters and segmental duplication might lead to scattered family members [43]. Only one tandem duplication cluster (VvBBX11/VvBBX22) in VvBBX gene family was identified on grapevine chromosome 12. Then, five pairs of duplicated segments (VvBBX7/VvBBX8, VvBBX10/VvBBX11, VvBBX13/VvBBX14, VvBBX14/VvBBX15, and VvBBX15/VvBBX16) in VvBBX gene family were identified within the grapevine genome (Fig. 2). The result suggested that segmental duplication events may be more important than tandem duplication in the expansion of the VvBBX gene family in grapevine. Furthermore, we also calculated the value of Ka/Ks of segmental genes’ pairs, which could be used as an indicator for the selection pressure of a gene during evolution. Our results showed that all the Ka/Ks values were less than 1, indicating that the *VvBBX* genes primarily evolved under the influence of purifying selection (Additional file 6: Table S1).

To better insight into the evolutionary relationships of the *VvBBX* genes, the intron/exon structures were investigated by aligning the cDNA sequences and corresponding genomic DNA sequences. As shown in Additional file 7: Figure S6, the number of exons varied from 1 to 5. *VvBBX13* and *VvBBX14* contained the highest amounts of exons (5), and *VvBBX22* and *VvBBX24* only had one exon among the *VvBBXs*. Additionally, the highly similar gene structure was identified in the same group of the *VvBBX* genes. For example, all three *VvBBXs* in the group I contained two exons, and most members within the group II had four exons except for *VvBBX9* (Additional file 7: Figure S6). These results suggested that exon-gain or -loss had occurred during the evolution of the VvBBX gene family and gene structure reveal the evolutionary relationship of VvBBXs.

### Identification of *cis*-elements in the promoters of *VvBBX* genes

To better understand the transcriptional regulation and the gene function of *VvBBX*, the *cis*-elements in the promoter regions of the *VvBBX* (2 kb of genomic DNA sequence upstream of the translation start site) were used to search the PlantCARE database (Additional file 8: Figure S7, Additional file 9: Table S2). As expected, CAAT-box and TATA-box, the conventional promoter elements, were found in all the *VvBBX* promoters. A series of *cis*-elements involved in plant growth and development, phytohormone responses and stress responses were identified (Additional file 8: Figure S7, Additional file 9: Table S2). As shown in Fig. 4, the CAT-box involved in meristem expression was identified in the promoters of 12 *VvBBX* genes. The zein metabolism regulation element (O2 site) was found in 10 *VvBBX* genes. Additionally, the seed-specific regulation element (RY element) and endosperm expression regulation element (GCN4_motif) were also found in the promoters of the *VvBBX* genes. Among the cis-acting elements involved in hormone responses, the abscisic acid responsive element (ABRE), ethylene responsive element (ERE), the MeJA responsive element (CGTCA-motif and TGACG-motif) and gibberellin-responsive element (GARE-motif, P-box and TATC-box) were found in the promoters of 21, 18, 17 and 12 *VvBBX* genes, respectively. Salicylic acid responsive element (TCA-element) and auxin-responsive element (AuxRR-core and TGA-element) were also observed in 9 and 5 *VvBBX* genes (Additional file 8: Figure S7, Additional file 9: Table S2). In stress-related responses elements, ARE, which was the most abundant element and involved in anaerobic induction, was found *VvBBX* genes. Some stresses-related (low-temperature, drought and wound) *cis*-acting elements were also found in the promoter regions of the *VvBBX* genes (Additional file 8: Figure S7, Additional file 9: Table S2). In addition, a number of light response elements were also found in the promoter regions of VvBBX, including Box 4, G-Box, G-box, GATA-motif, GT1-motif, I-box, MRE, TCCC-motif and TCT-motif.

### The organ-specific expression patterns analysis of *VvBBX* genes in grapevine

**Fig. 3:**
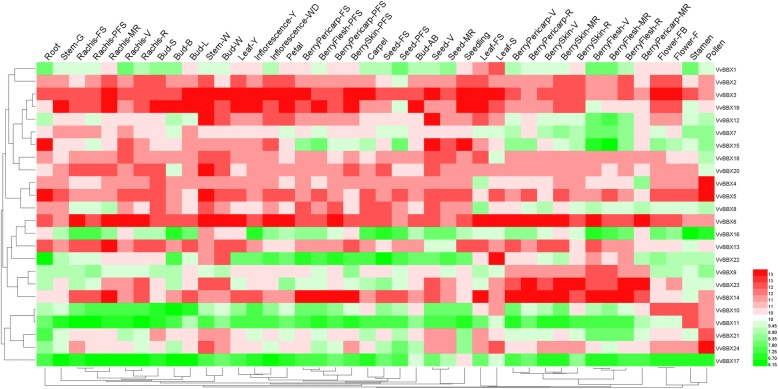
Expression profiles of the grapevine VvBBX genes in different organs, tissues and developmental stages. Data were normalized based on the mean expression value of each gene in all tissues analysed. Genes were hierarchically clustered based on average Pearson’s distance metric and ‘average linkage’ method. Red and green boxes indicate high and low expression levels, respectively, for each gene. Bud-AB, bud after burst; Bud-B, Bud burst; Bud-W, winter bud; Bud-L, latent bud; Bud-S, bud swell; Flower-F, flowering; Flower-FB, flowering begins; FS, fruit set; Inflorescence-Y, young inflorescence with single flowers separated; Inflorescence-WD, well-developed inflorescence; Leaf-FS, mature leaf; Leaf-S, senescing leaf; Leaf-Y, young leaf; MR, mid-ripening; R, ripening; PFS, post fruit set; Stem-G, green stem; Stem-W, woody stem; V, véraison

In order to investigate the putative roles of the *VvBBX* genes in grapevine development, the organic-specific expression patterns of *VvBBXs* were analyzed in the *V. vinifera* cv. Corvina global gene expression atlas from the GEO DataSets (GSE36128), which contained 42 various organs/tissues at different developmental stages obtained by microarray analysis (Fig. 3, Additional file 10: Table S3). As shown in Fig. 3, some *VvBBX* genes exhibited similar expression profiles in different organs/tissues, while other *VvBBXs* showed tissue-specific transcript accumulation patterns, potentially suggesting the functional divergence of *VvBBX* genes during grapevine growth and development. For example, four *VvBBX* (*VvBBX2*, *3*, *5* and *6*) genes were ubiquitously high expressed in nearly all tissues tested, whereas *VvBBX16* and *VvBBX17* were expressed at a very low level in all tested tissues.

Some *VvBBX* genes showed a very high level in specific organs/tissues. For example, three *VvBBX* (*VvBBX4*, *21* and *24*) genes displayed higher expression level in pollen than other tissues, implying that they were important proteins for signaling in grapevine pollen growth and development. *VvBBX1* and *VvBBX22* exhibited high levels of expression in senescing leaf, which suggested that they might be involved in leaf senescing. *VvBBX20* showed relatively high expression level in woody stem (Stem-W), indicating involvement in lignification of xylem and phloem during secondary growth of woody tissues. *VvBBX15* was preferentially expressed in seedling, root, and seed (seed V), which suggested *VvBBX15* played important roles in three tissues. Furthermore, two pair *VvBBX* in the segmental duplications showed similar expression patterns. For example, *VvBBX7* and *VvBBX8* with similar gene structure and common motif composition had low expression abundances in berries. *VvBBX10* and *VvBBX11* showed relatively high expression level in the stamen. (Fig. 3, Additional file 10: Table S3). Remarkably, *VvBBX6*, *VvBBX14,* and *VvBBX23* were highly expressed in berries, which indicated that these genes may play an important role in berry development and ripening. These results aroused us to investigate the expression patterns of *VvBBX* genes during different fruit development and ripening stages.

### Expression patterns of *VvBBX* genes during berry developmental and ripening

**Fig. 4:**
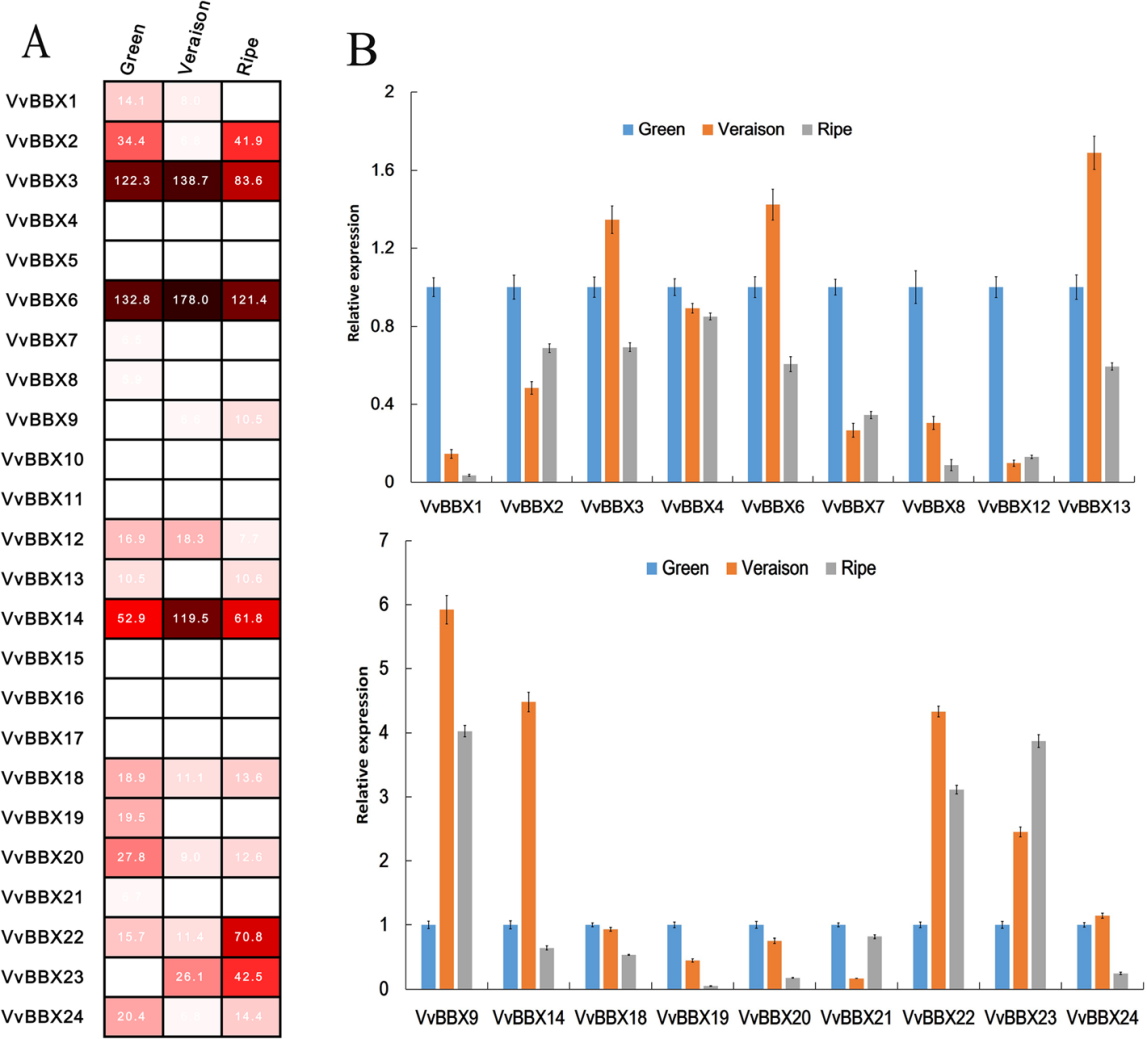
Expression profiles of the grapevine VvBBX genes during three fruit developmental stages. **a** Hierarchical clustering of the transcript accumulation profiles of 24 VvBBX genes during three berry developmental stages. **b** RT-qPCR transcript analysis of 18 selected VvBBX genes at three berry developmental stages. Berries from 3 year old ‘Fujiminori’ grapevine trees were sampled in triplicate at the fruit expanding (40DAF or DAF40), veraison (65DAF or DAF65), and ripe (90DAF or DAF90) stages throughout the growing season. The experiments were repeated three times and provided consistent results

To understand the potential function of *VvBBX* genes in berry development and ripening, the transcript expression patterns of 24 *VvBBX* genes were investigated during three fruit developmental stages in grapevine using the expression profiles from the NCBI Gene Expression Omnibus (GEO) DataSets (GSE77218). Different members of the *VvBBX* genes showed distinct transcript accumulation patterns during different fruit developmental stages. As shown in Fig. 4a, *VvBBX3*, *VvBBX6,* and *VvBBX14* showed relatively high expression levels during the ripening process, whereas six *VvBBX* genes (*VvBBX5*, *10*, *11*, *15*, *16* and *17*) were almost undetectable during different berry developmental stages (Fig. 4a, Additional file 11: Table S4). 11 *VvBBX* genes were down-regulated expression patterns, while three *VvBBX* genes displayed up-regulated expressions during fruit developmental (Fig. 4a, Additional file 11: Table S4). For example, the expression level of *VvBBX1* was moderate and gradually decreased from veraison till to ripe stage. Similar to *VvBBX1*, but to a lesser extent, *VvBBX18*, *VvBBX19,* and *VvBBX20* had the highest expression at green fruit stage. *VvBBX22* and *VvBBX23* exhibited a significant increase in expression during berry development and reached their picks at the ripening stage (Fig. 4a, Additional file 11: Table S4).

In order to validate the transcript abundance of *VvBBX* genes in the various developmental stages of the berry by microarray data, qRT-PCR analysis of all 18 detectable *VvBBX* genes was further performed at three berry development stages. As was expected, qRT-PCR results were highly consistent with the RNA-Seq data except for *VvBBX13* and *VvBBX24* (Fig. 4b). For example, the expression of *VvBBX1* and *VvBBX23* were dramatically decreased and increased during berry developmental and ripening, respectively. *VvBBX18*, *VvBBX19,* and *VvBBX20* also depicted the highest expression at the green fruit stage (Fig. 4b). All these results were consistent with the data from RNA-Seq data. However, the expression profiles of *VvBBX13* and *VvBBX24* did not correspond with RNA-Seq data. *VvBBX13* were highly expressed in ripe stage from RNA-Seq data, whereas the qRT-PCR result showed the highest expression in the veraison stage. *VvBBX24* had a relatively high expression in veraison berry from qRT-PCR analysis, whereas the RNA-Seq data showed the highest expression in the green berry stage (Fig. 4b). All these results implied that some *VvBBX* genes showed different expression patterns and might play essential roles during fruit development and ripening.

**Fig. 5:**
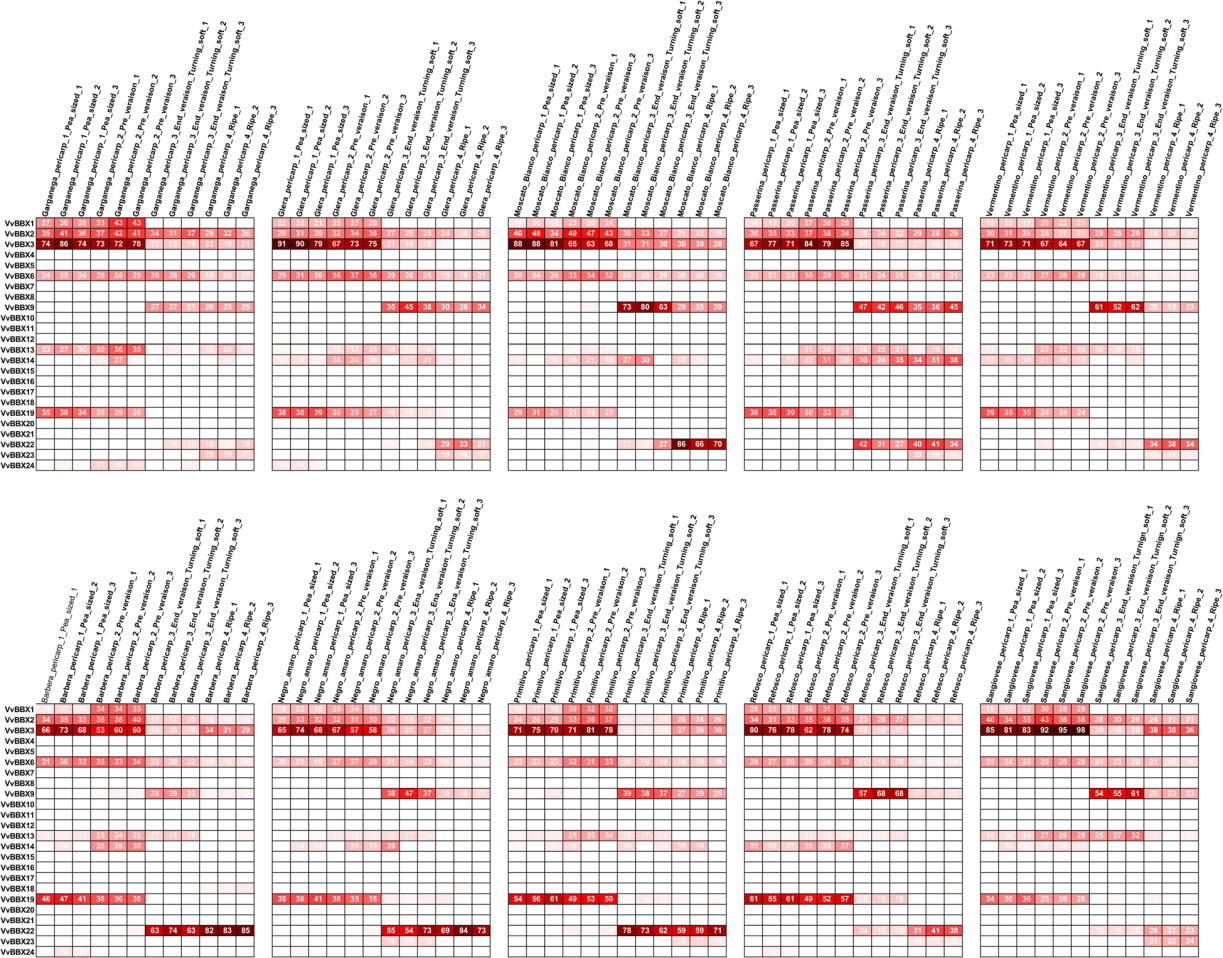
Expression profiles of the grapevine VvBBX genes in 10 different grapevine varieties at four berry developmental stages. Berries were sampled in triplicate at four developmental stages, the pea-sized berry stage at 20d after flowering, the berries beginning to touch stage just prior to veraison (Pre_veraison), the berry-softening stage at the end of veraison (End_veraison), and the fully ripe berry stage at harvest

To provide more information on the berry developmental and ripening functions of *VvBBX* genes in grapevine, we investigated their transcript accumulation patterns among 10 different grapevine varieties by using microarray data from the NCBI GEO DataSets (GSE62744 and GSE62745), which consists of four different fruit developmental stages (the pea-sized berry stage at 20d after flowering, the berries beginning to touch stage just prior to veraison, the berry-softening stage at the end of veraison, and the fully ripe berry stage at harvest [44]. As shown in Fig. 5, the transcript accumulation profiles of *VvBBX3* and *VvBBX6* remained relatively strong but decreased gradually throughout grapevine fruit ripening, which was corresponded with the data from RNA-Seq data. *VvBBX9* and *VvBBX22* were preferentially expressed in End_veraison and ripe stage, and the similar expression pattern was also observed from RNA-Seq data. On the contrary, *VvBBX1* and *VvBBX19* were higher expression level in pea-sized berry and Pre_veraison stage and rapidly down-regulated during ripening, which was agreed with the data from RNA-Seq and qRT-PCR analysis (Fig. 4). Additionally, 12 *VvBBX* genes exhibited slight or no expression in fruit among all 10 grapevine varieties. All these results indicated that some *VvBBX* genes maybe play multiple important roles in grapevine fruit development.

### *VvBBX* genes in response to exogenous ABA, ethylene, GA_3_ and CPPU hormones

**Fig. 6:**
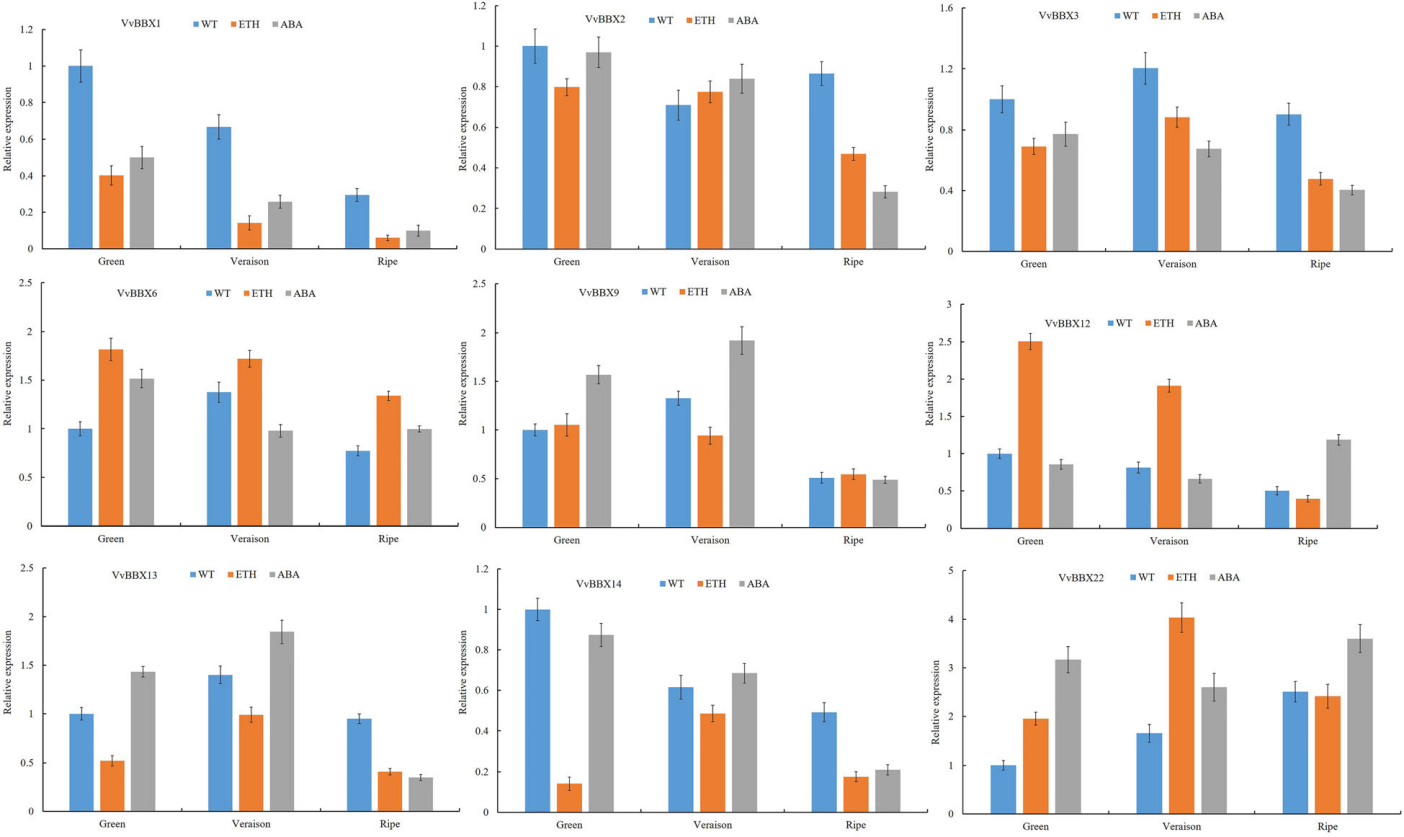
Expression profiles of grapevine BBX genes in response to ABA and ethylene treatment. Data were normalized to the expression level of the actin gene. The mean value was calculated from three independent replicates. Vertical bars indicate the standard error of the mean

Plant hormones are originally characterized as regulators in growing and developmental processes, simultaneously, the role of BBX proteins in hormonal signaling pathways is scarce in grapevine. To reveal the potential roles of the *VvBBX* genes in response to (abscisic acid) ABA and ethylene treatments, qRT-PCR was used to analyze the expression of the *VvBBX* genes, which was relatively high expression during grapevine berry ripening. As shown in Fig. 6, the expression of *VvBBX1* was significantly inhibited by pre-veraison ABA and ethylene treatment throughout the entire berry development and ripening period, indicating that *VvBBX1* play a negative role in the regulation of ripening (Fig. 6). Similarity, *VvBBX2*, *VvBBX3* and *VvBBX13* seemed to be suppressed at ripening stage, implying that these two genes in grapevine might be act as a negative regulator of fruit ripening. *VvBBX6* and *VvBBX9* were slight increase by ethylene and ABA treatment, respectively. *VvBBX12* was up-regulated at green and veraison stage and *VvBBX14* was down-regulated at green and ripe stage after exogenous ethylene treatment (Fig. 6), indicating that VvBBX12 and VvBBX14 might have opposite function in response to ethylene signaling pathway. It is worth noting that the transcript level of *VvBBX22* was significantly upregulated at green and veraison stage by ABA and ethylene treatment (Fig. 6), indicating *VvBBX22* might play a positive role in response to ABA and ethylene treatments.

Currently, GA_3_ and forchlorfenuron (CPPU) commonly applied to increase fruit weights, produce seedless grapes and inhibit russet development [45]. Therefore, we further investigated the potential roles of the *VvBBX* genes in response to GA3 and CPPU by previous RNA-seq data [45]. As shown in Additional file 12: Table S5, the expression of *VvBBX7*, *VvBBX8* and *VvBBX12* was significantly increased, whereas *VvBBX21*, *VvBBX22* and *VvBBX24* was significantly decreased by GA3 and CPPU treatment (Additional file 12: Table S5).

### Expression patterns of *VvBBX* genes under different abiotic stresses

**Fig. 7:**
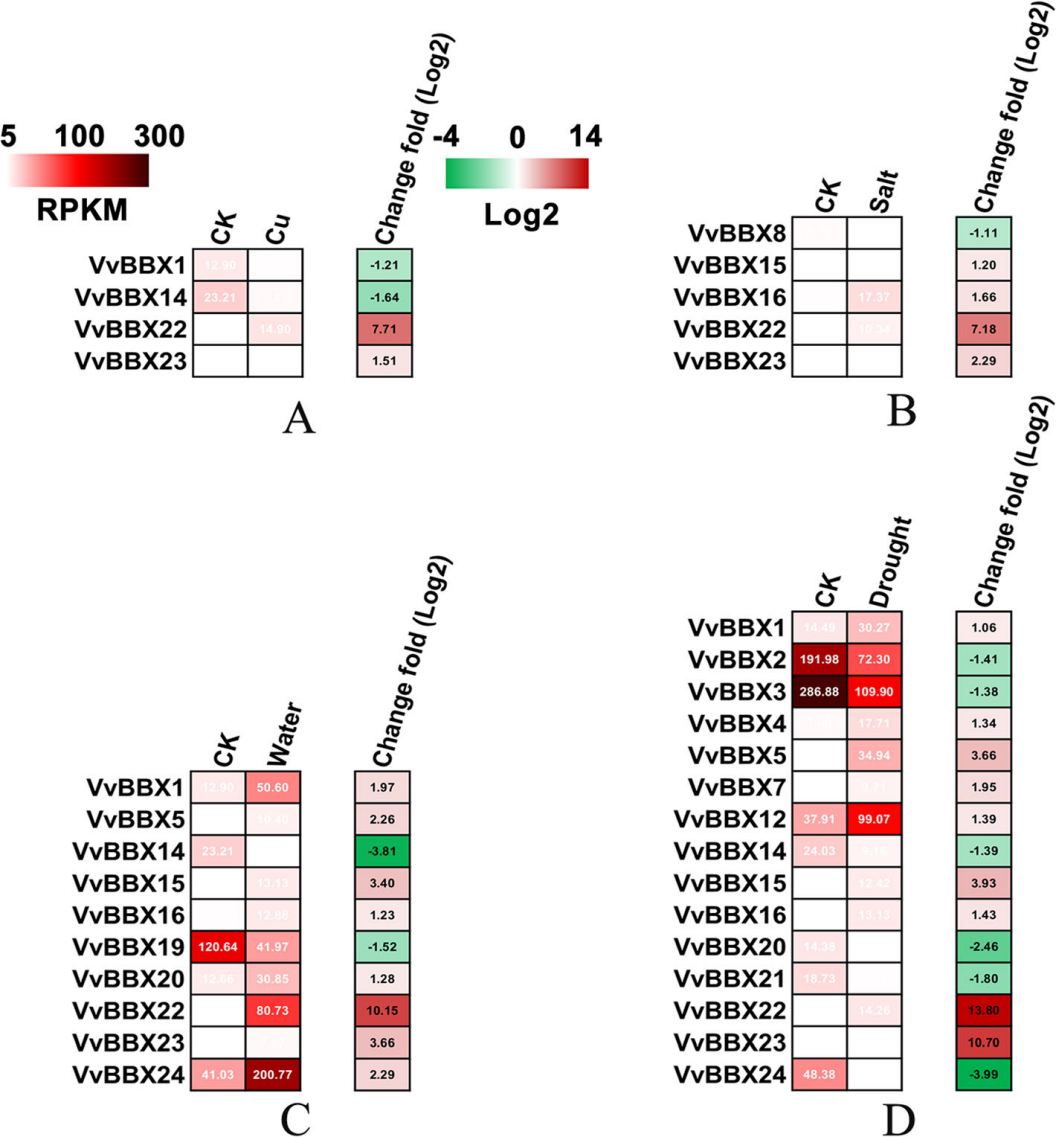
The expression of VvBBXs under different abiotic stresses. **a-d**, Hierarchical cluster displaying the differentially expressed VvBBX genes under Cu, salt, waterlogging and drought treatments. Data were obtained by RNA Sequencing and were expressed as Reads Per Kilobase of exon model per Million mapped reads (RPKM). The differentially expressed data were log2 transformed with R software. Blocks with green colors indicate decreased and red ones indicate increased transcription levels

Copper (Cu), salt, waterlogging and drought are common abiotic stresses of vineyards. To understand the potential functions of the *VvBBX* genes in responses to these different environmental stresses, we collected RNA-seq data sets for grapevines subjected to the above four stresses (drought stress, 20 days, SRP074162, waterlogging stress, 48 h, SRP070475, salt, 48 h, see Additional file 2 in Int. J. Mol. Sci. 2018, 19, 4019, copper stress, 24 h, see Additional file 9: Table S2 in Sci. Rep. 2015, 5, 17,749). Overall, the *VvBBX* genes responded to waterlogging and drought stress to a greater extent than to Cu and NaCl treatment (Fig. 7, Additional file 13: Table S6). Among them, two *VvBBXs* (*VvBBX22* and *VvBBX23*) were significantly induced against the Cu-treated sample and two *VvBBXs* (*VvBBX1* and *VvBBX14*) were down-regulated by Cu stress (Fig. 7, Additional file 13: Table S6). Under salt stress, four *VvBBX* genes were up-regulated and only *VvBBX8* was down-regulated. In contrast, 10 *VvBBX* genes were identified as differentially expressed genes under waterlogging stress, of which, only *VvBBX14* and *VvBBX19* were down-regulated, and the remaining genes were up-regulated (Fig. 7, Additional file 13: Table S6). In response to drought stress, nine *VvBBX* genes showed an increased expression pattern and six *VvBBXs* were more or less reduced (Fig. 7, Additional file 13: Table S6). Notably, four *VvBBXs* (*VvBBX1*, *14*, *15* and *16*) responded to at least three treatments. Interestingly, *VvBBX22* was strongly induced at least 7-fold by all four types of stresses, suggesting that *VvBBX22* might be a candidate gene for mitigating abiotic stresses. Similar to *VvBBX22*, but to a lesser extent, *VvBBX23* was also induced dramatically by various environmental stresses (Fig. 7, Additional file 13: Table S6).

### Subcellular localization of grapevine BBX proteins

**Fig. 8:**
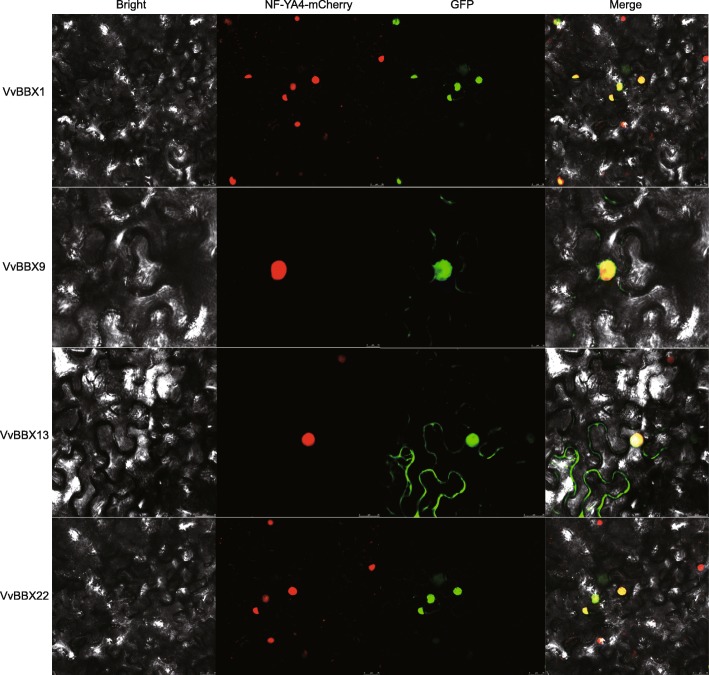
Subcellular localization of four GFP-fused VvBBX proteins. The four VvBBX-GFP fusion proteins (VvBBX1-GFP, VvBBX9-GFP, VvBBX13-GFP and VvBBX22-GFP) were transiently expressed in tobacco leaves and observed by fluorescence microscopy 72 h later. Nucleus were visualized by co-transformation with an mCherry-labelled nuclear marker ((NF-YA4-mCherry)

The nuclear localization of transcription factors is very important for its regulatory function. Previous studies showed that BBX proteins were predominantly located on the nucleus, such as PbBBX4, PbBBX5, and PbBBX13 in pear [40]. The subcellular localization of VvBBX proteins was firstly predicted by WoLF PSORT (Table 1). Seventeen of them had a high probability to be located in the nucleus. Four genes (VvBBX1, 9, 13 and 22) which were specifically expressed in fruit, were selected for a transient expression assay in *Nicotiana benthamiana* epidermal cells. As indicated in Fig. 8, green fluorescence signals from the expressed fusion VvBBX1-GFP, VvBBX9-GFP, VvBBX13-GFP, and VvBBX22-GFP were specifically distributed within the nucleus as confirmed by a mCherry-labelled nuclear marker (NF-YA4-mCherry). Notably, VvBBX13-GFP were also localized in the cytoplasm (Fig. 8), which was inconsistent with the prediction result (Table 1). These results suggested that VvBBX1, VvBBX9, and VvBBX22 were nuclear proteins, and consistent with the previous results [39, 40].

## Discussion

The BBX protein is one of the important transcription factors, which play an important role in regulating plant growth and development [4]. To date, the features and functions BBX gene family have been identified in several plant species, such as *Arabidopsis* [3], rice [46], tomato [39], pear [40] and apple [41]. Although a comprehensive analysis of the *BBX* gene family in grapevine during growth, berry development, and stress response has not been studied so far. In this study, we performed a genome-wide analysis of the *BBX* genes in grapevine by investigating their linkage group organization, phylogenetic relationships, gene structure, duplication events, cis-acting elements, gene expression profiles in different tissues and developmental stages, and under various stress treatments. Genome-wide analysis of the BBX genes in grapevine will set a foundation for further functional studies of this gene family for the molecular cloning of grapevine.

### Evolution of the grapevine BBX gene family

The presence of BBX genes in the genome of different species from algae to monocots and dicots clearly suggests an ancient origin [4, 47]. A total of 24 *VvBBX* genes were identified from the grapevine genome, and the number of *VvBBX* genes was much smaller than that from other plants, such as 64 for *Malus domestica* [41], 32 for *Arabidopsis* [3], 30 for rice [46], and 29 for tomato [39]. The difference might be due to the variable state of paralogous genes in these genomes. For example, only 5 paralogous gene pairs were found in grapevine, while 22 paralogous and 9 paralogous gene pairs from segmental duplication events were identified in apple and rice, respectively. This conclusion was further supported by the previous study in *D. officinale* (19 members) and *P. equestris* (16 members), including only two DoBBXs paralogous and two PeBBXs paralogous gene pairs, respectively [48]. Furthermore, the composition of the *BBX* gene in different subgroups was also different among species (Additional file 14: Figure S8). In grapevine, the numbers of BBX members with two tandem B-BOXes plus the CCT domain, two tandem B-BOXes, Box 1 plus CCT, and B-Box 1 only were 8, 9, 2 and 5, respectively (Additional file 14: Figure S8). The corresponding numbers were 13, 8, 4, and 7 in *Arabidopsis* [3], while 8, 10, 5 and 6 were in tomato [39]. These results demonstrated that BBX genes of different species might share a common ancestor, and underwent an independent expansion after the divergence of the monocots and the dicots [40].

Sequence alignment and phylogenetic analysis showed that all VvBBXs were classified into five subgroups, which are consistent with those of *Arabidopsis*, tomato and pear BBXs [3, 39, 40]. All *VvBBX* genes from subgroup I, II and IV had two B-BOX domains in grapevine, except for *VvBBX11* (Fig. 1a). In contrast to animal BBX with two different types of B-BOXs, the amino acid sequences of the two B-BOX domains in grapevine were more conservative and retained the same topology [49]. Most green algae had only a single B-box domain. However, two B-box domains were found in the unicellular green alga *Chlamydomonas*, implying that the B-box duplication event has taken place in some cases much before the colonization of land plants [4, 49, 50]. The rapid expansion of BBX proteins during evolution, and the fact that they are highly conserved across the plant kingdom indicates that BBX proteins might play important roles in the adaptation of land plants [4, 50].

Tandem duplication and segmental duplication events play a crucial role in the expansion of gene family members during plant evolution [43]. To further elucidate the expansion mechanism of the *VvBBX* gene family in grapevine, both the tandem and segmental duplication events were analyzed. The results demonstrated that eight *VvBBX* genes (*VvBBX7*/*VvBBX8*, *VvBBX10*/*VvBBX11*, *VvBBX13*/*VvBBX14*, *VvBBX14*/*VvBBX15*, and *VvBBX15*/*VvBBX16*) were identified to participate in the segmental duplication (Fig. 2). Moreover, only one gene pair VvBBX11/VvBBX22 showed tandem duplication (Fig. 2), indicating that segmental duplication was more common in *VvBBX* genes. Taken together, these results suggested that both segmental and tandem duplications contributed to the expansion of the grapevine *VvBBX* gene family during their evolution. The same potential mechanism of gene family evolution was also identified in the SBP-box and WRKY gene family in grapevine [51, 52]. Furthermore, the value of *K*a/*K*s of segmental gene pairs was also calculated. Generally, Ka/Ks ratio greater than 1, equal to 1, and less than 1 represents positive selection, neutral selection, and negative selection, respectively. Remarkably, the Ka/Ks ratios of all grapevine gene pairs were less than 1, indicating that these gene pairs have been experiencing a markedly purifying selection during their evolution.

### Potential roles of *VvBBX* genes in plant growth and development

Accumulating studies have shown that *BBX* genes are involved in multiple aspects of plant growth and development, such as seedling photomorphogenesis, shade avoidance, chlorophyll accumulation, and flower induction [4]. The expression pattern of 24 *VvBBX* genes in 42 different grapevine tissues during different developmental stages were examined using an expression atlas of *V.vinifera* (cv. Corvina) [53]. The gene expression analysis indicated that some *VvBBX* genes can be classified together according to their expression abundance in tissue-specific response of grapevine, probably reflecting their participation in a common metabolic and/or developmental process. In addition, several *VvBBX* genes might have a unique function in specific developmental stages.

Previous studies have also shown that *BBX* genes are involved in pollen tube growth. For example, the expression of *PbBBX5* increased significantly in the pear pollen tubes, suggesting that it may play a critical role in pear pollen tube growth [40]. Moreover, *VvBBX4*, an ortholog of *PbBBX5* showed relatively high transcriptional activity in the pollen tube, indicating its potential involvement in the pollen tube growth and/or senescence. Likewise, *VvBBX24* might play a similar role in grapevine, as it was also highly expressed in the pollen tube. *BBX* genes participate in the photoperiod pathway of flowering. In *Arabidopsis*, *BBX6/COL5* promotes flowering under short-day by enhancing the expression of *FT* [54], whereas *BBX7/COL9* negatively regulates flowering by repressing the expression of *CO* and *FT* under long day [23]. *BBX32/EIP6* represses flowering probably in a CO-independent manner under long-day [55]. *VvBBX10* and *VvBBX11* were expressed at a higher level in floral tissues, signifying their putative role in the regulation of flower development. Taken together, our results proposed that *VvBBX* genes were not only involved in the development of pollen tubes, but also in the regulation of flower development.

AtBBX21 (also known as SALT TOLERANCE HOMOLOG 2, STH2) interacts with ELONGATED HYPOCOTYL 5 (HY5), which is considered as a key signaling regulator in photomorphogenesis, and positively regulates seedling photomorphogenesis [56]. *AtBBX21*, which shows expression in dry and germinative seeds, is also involved in ABA signaling and control seed germination [57]. In our findings, *VvBBX15*, the closest homolog of *AtBBX21* in grapevine, showed significantly higher relative expression level in seedling and veraison seed, indicating that the *VvBBX15* might be involved in seedling photomorphogenesis and seed development. On the contrary, *AtBBX24* (*STO*) and *AtBBX25* (*STH*) show opposite functions to *AtBBX21* and suppress seedling photomorphogenesis [29, 58]. *VvBBX19*, which is closely related to *AtBBX24* and *AtBBX25*, also expressed at high levels in the seedling. The result speculated that the *VvBBX19* gene might be involved in grapevine photomorphogenesis and displayed opposite functions with *VvBBX15*. However, *VvBBX19* showed more widespread and less tissue-specific transcript accumulation patterns, such as in buds, leaves, floral parts, and fruits. These results indicated that *VvBBX19* genes might participate in regulatory functions during multiple growth and developmental stages. Additionally, *CmBBX22* plays an important role in delaying leaf senescence and enduring drought severity in chrysanthemum [59]. The expression of *VvBBX22* was significantly higher in senescent leaf than other tissues, suggested that *VvBBX22* is likely to play similar roles as *CmBBX22* in regulating leaf senescence in grapevine. Taken together, all the *VvBBXs* were expressed at most tissues and developmental stages tested, which revealed that *VvBBX* genes can play multiple important roles in various developmental and biological processes.

### *VvBBX* is likely to play important roles during grapevine berry development and ripening

Grape berry development and ripening is a complex physical and biochemical process that is controlled by various transcriptional networks and regulatory proteins, such as MYB, bHLH and MADS-box [60]. There are growing evidences that *BBX* genes are involved in anthocyanin accumulation and berry ripening [56, 61, 62]. For example, *AtBBX22* directly promotes the expression of the *production of anthocyanin pigment 1* (*PAP1*) and anthocyanin biosynthesis genes by interacting with *AtHY5* [56, 61]. Mutation of *AtBBX22* displays lower anthocyanin accumulation by down-regulation of *PAP1* expression, suggesting that *AtBBX22* modulates anthocyanin synthesis by regulating the expression of MYB genes [10, 61]. Studies in apple proposed that *MdBBX22* (*MdCOL11*) involves in MdHY5-mediated signal transduction network and positively regulates anthocyanin biosynthesis in apple peel [36]. On the contrary, *MdBBX54* (*MdCOL4*) suppresses anthocyanin accumulation in apple skin by inhibiting the expression of *MdANS* and *MdUFGT*, which encode genes in the anthocyanin biosynthetic pathway. Moreover, *MdBBX54* indirectly inhibits the expression of *MdMYB1* by interacting with MdHY5 [63]. In rice, *OsBBX14* also interacts with the *OsHY5* and regulates anthocyanin biosynthesis [64]. Furthermore, transcript accumulation of *MaCOL1* in banana pulp markedly increased during natural or ethylene-induced fruit ripening, indicating that *MaCOL1* is a transcription activator and might be associated with the ripening of banana fruit [62]. *PaBBX28* is highly co-expressed with *ANS*, *CHI*, *F3H*, and *F3′H*, which are deliberated as four vital genes for anthocyanin biosynthesis, suggesting that the BBX transcription factors are involved in anthocyanin biosynthesis in sweet cherry [65].

In grapevine, *VvBBX18* and *VvBBX19* share high homology with the *AtBBX22* and *MdBBX54*, showed high expression levels in green berries (Fig. 4), indicating their potential role at the early stage of grape berry development. *VvBBX1* exhibits a similar high expression with *VvBBX18* and *VvBBX19* in green berries, suggesting that *VvBBX1* may likely to perform a function similar to *VvBBX18* and *VvBBX19*. Interestingly, protein interaction network by STRING V11 revealed that both *VvBBX18* and *VvBBX19* showed interaction with the *VvHY5* (Additional file 15: Figure S9 and Additional file 16: Table S7). These results are consistent with the previous studies in *Arabidopsis* and apple [36, 61, 63] and signifies that *VvBBX18* and *VvBBX19* can regulate anthocyanin accumulation or berry development by MdHY5-mediated signal transduction network. *VvBBX14*, the homologs of *MdBBX22*, exhibited the highest expression at veraison stage, implying its role in anthocyanin accumulation and berry ripening (Figs. 4, 5). Additionally, *VvBBX9*, *VvBBX22,* and *VvBBX23* were preferentially expressed in ripe berries, indicating that these three genes may be involved in grapevine berry ripening process.

### Potential roles of *VvBBX* in response to various phytohormone in grapevine

Although grapevine berries have been classified as non-climacteric fruits, several hormones including ABA and ethylene may involve in the control of ripening in grape berry. It is well known that ABA promote grape berry ripening, and ABA content in berry shows a strong increase at the end of the color turning period and during the initial stages of ripening [66]. Exogenous ABA treatment causes an increase in berry weight, a decrease in titrable acidity, and an increase in total anthocyanin content [60]. Futhermore, ethylene can affect the physiological processes during maturation of grapevine, including berry expansion and anthocyanin accumulation [67]. Exogenous ethylene treatment stimulates berry coloration and enhances the expression of genes related to the anthocyanin biosynthesis [68]. Ethylene application at veraison also leads to a berry expansion by increasing the expression of water exchange and cell wall structure genes [69]. At present, the role of *VvBBX* genes in the ABA and ethylene signaling pathways is still poorly studied. In previous studies, four tomato BBX genes were induced by ETH, and all of them had the ethylene-responsive cis-element (ERE) in their promoters [39]. In *Arabidopsis*, *AtBBX21* coordinates with HY5 and ABI5 on the ABI5 promoter and that these transcriptional regulators work in concert to integrate light and ABA signaling [57]. In our study, the expression of *VvBBX1* was significantly decreased during the whole berry ripening period and three other *VvBBX* genes (i-e., *VvBBX2*, *VvBBX3,* and *VvBBX13*) were also suppressed at ripening stage by pre-veraison ABA and ethylene treatment, implying that these genes might be act as a negative regulator of fruit ripening in grapevine. By contrast, *VvBBX22* was upregulated expression after exogenous ABA and ethylene treatment, suggesting that *VvBBX22* can positively regulate fruit ripening in grapevine. All these findings indicated that these *VvBBX* genes might be involved in multiple hormone signaling as transcriptional regulators to modulate berry development and ripening.

In addition, *VvBBX* genes might be attributed to grape berry expansion and seedlessness. Multiple *VvBBX* genes participated in response to GA_3_ and CPPU treatment, which were commonly applied to increase berry size and inhibit russet development in vineyard. For example, the expression of *VvBBX7*, *VvBBX8* and *VvBBX12* was significantly increased, whereas *VvBBX21*, *VvBBX22* and *VvBBX24* was significantly decreased by GA_3_ and CPPU treatment (Additional file 12: Table S5). The current results indicated that the majority of VvBBX genes detected herein were up-regulated or down-regulated following the GA_3_ and CPPU treatment, demonstrating that BBX transcription factors might have opposite functions in grape berry expansion and seedlessness. Interestingly, the expression of *VvBBX22* seemed to be responding to multiple hormone signaling, including ABA, ethylene, GA3, and CPPU, implying that *VvBBX22* regulated multiple aspects of grapevine fruit ripening by modulating multiple hormone signaling pathways.

### Potential roles of *VvBBX* genes in response to different abiotic stress in grapevine

Transcriptional regulation of stress-responsive genes is an important part of the plant response to a series of abiotic and biotic stresses. Transcription factors are master regulators that control the expression of gene clusters by binding the cis-acting element in the promoter regions of target genes. Various abiotic stresses, including Cu, salinity, waterlogging, and drought have a negative impact on grapevine growth and development. Previous studies have shown that *BBX* genes are involved in various abiotic stresses response [39, 41]. In this study, a series of stress-responsive cis-acting elements, such as ARE, DRE, MBS, and TC-rich, frequently occurred in the promoter regions of *VvBBX* genes (Additional file 9: Table S2), and are involved in drought, salt, and waterlogging. All 24 *VvBBX* genes possessed at least one of the stress-responsive *cis*-acting element, indicating their potential functions in response to abiotic stresses (Additional file 9: Table S2). In our findings, eight *VvBBX* (*VvBBX1*, *5*, *15*, *16*, *20*, *22*, *23*, *24*) genes were induced against waterlogging stress, and all of them contained ARE elements in their promoters. Furthermore, there were four, five, ten, and fifteen *VvBBX* genes showing potential involvement in Cu, salt, waterlogging, and drought, respectively. The results suggested that the majority of *VvBBX* genes were induced or repressed at varying degrees depending on stress treatments. In addition, both *VvBBX15* and *VvBBX16* were co-expressed (upregulated) in salt, waterlogging and drought treatments (Fig. 7), indicating that these two genes may integrate different stress signals. The expression of *VvBBX1* was enhanced by waterlogging and drought stress but was declined under Cu stress, suggesting that *VvBBX1* might have different mechanisms to maintain protection against various abiotic signals. In particular, the expression of *VvBBX22* was up-regulated by 7–13 fold under four abiotic stresses (Fig. 7), indicating *VvBBX22* might play a vital role in response to multiple abiotic stress networks. Our result is consistent with the high expression of *VvBBX22* in senescent leaf and implied that *VvBBX22* might under various abiotic stresses. Characterization of *VvBBX* genes in response to different abiotic stresses will greatly improve our understanding of the functions and the crosstalk that occurs among different abiotic stresses signaling pathways.

## Conclusions

In this study, 24 *VvBBX* genes were identified in grapevine, and the systematic and comprehensive analysis of the *VvBBX* gene family was performed, including conserved domain, phylogenetic relationship, gene structure, chromosome location, gene duplication, cis-acting elements, and expression pattern analysis. Numerous cis-acting elements were found in the VvBBX promoter sequences, indicating that *VvBBX* genes were involved in complex regulatory networks controlling development and responses to abiotic stresses. The transcription of *VvBBX* in different tissues and developmental stages and under various stress conditions indicated that *VvBBX* may have various functions in grapevine growth and development. Transcriptome and qRT-PCR analysis revealed *VvBBX* genes might play important roles in fruit developmental and ripening stages by modulating multiple hormone signaling pathways. Taken together, genome-wide analysis of the *VvBBX* will provide a solid foundation for functional analyses of BBX genes in grapevine, and further study on several BBX genes is undergoing to understand their biological functions.

## Methods

### Identification of BBX genes in the grapevine genome

Two different procedures were used to identify and interpret the BBX gene in grapevine as previous reports [70]. First, all the Arabidopsis BBX protein sequences downloaded from the Arabidopsis information source (TAIR) database (http://www.arabidopsis.org) were used as queries, and grape genome database was screened by the blast p Program (E-value <1e-5). Second, the hidden Markov model (HMM) of b-box domain (pfam00643) was established by Pfam database (http://pfam.xfam.org/) and (http://genomes.cribi.unipd.it/grape/) was used to retrieve the grape genome database. Subsequently, the potential VvBBX genes in grapevine genome were further verified for the presence of the B-BOX domain by screening against the Pfam (http://pfam.sanger.ac.uk/), InterProScan (http://www.ebi.ac.uk/Tools/pfa/iprscan/) and SMART (http://smart.embl-heidelberg.de/) database. The molecular weights, isoelectric points (pI) and grand average of hydropathicity (GRAVY) of VvBBX proteins were calculated by the ExPasy website (https://web.expasy.org/protparam/). The subcellular locations of grapevine BBX proteins were predicted by WoLF PSORT (http://www.genscript.com/psort/wolf_ psort.html).

### Construction of VvBBXs phylogenetic tree and sequence alignment analysis

ClustalW program (version 2.1; http://www.clustal.org/) was used for multiple sequence alignment of 24 VvBBX proteins. A phylogenetic tree based on VvBBXs protein sequence was constructed by using MEGA5.0 program neighbor-joining method and bootstrap analysis (1000 replicates) [71]. Domains were identified with Pfam (http://pfam.xfam.org), InterProscan (http://www.ebi.ac.uk/interpro/search/sequence-search) and SMART (http:// smart.embl-heidelberg.de) programs. The motif logos of the VvBBXs were generated by submitting the sequences to the MEME website (http://meme.nbcr.net/meme/cgi-bin/meme.cgi).

### Chromosomal location, gene structure, and duplication analysis

All *VvBBX* genes were mapped to grapevine chromosomes based on physical positions at the Grape Genome CRIBI website (http://genomes.cribi.unipd.it/) and MapInspect software was used for drawing of the map. Accordingly, the cDNA sequences and its corresponding genomic DNA sequences of *VvBBX* members were obtained from grape genome, and then the coding sequence and its corresponding genomic sequence were compared by using GSDs software (http://gsds.cbi.pku.edu.cn) to determine the exon-intron organization [72]. Tandem duplicated genes were determined by detecting its physical locations on specific chromosomes and were identified as adjacent paralogous on a grape chromosome, with no more than one intervening gene. For joint analysis, download and analyze the joint blocks in the grape genome from the plant genome replication database [73]. For duplicate pairs, Ka (nonsynonymous substitution rate) and Ks (synonymous substitution rate) and evolutionary constraint (Ka/Ks) between paralogous pairs of VvBBX genes were calculated by ParaAT and KaKs_Calculator as previous report.

### *Cis*-element analysis for VvBBX gene promoter

The promoter sequence of 2000 bps upstream of each *VvBBX* genes coding regions were retrieved from CRIBI (http://genomes.cribi.unipd.it/). PlantCARE online program (http://bioinformatics.psb.ugent.be/webtools/plantcare/html/) was used to search for assumed *cis*-acting element [74].

### Expression profiles of VvBBXs in various organs and different berry developmental stages

The expression profile of *VvBBX* gene in a *V. vinifera* cv ‘Corvina’ (clone48) in different organs at different developmental stages was determined. Microarray data were attained from the NCBI gene expression omnibus (GEO) datasets under the series entry GSE36128 (https://www.ncbi.nlm.nih.gov/geo/query/acc.cgi?acc=GSE36128) [53]. Mean of expression value of each gene in all tissues of different organs were evaluated and graphically characterized using Multi Experiment Viewer (MeV) software [75]. Expression patterns of *VvBBX* genes in developmental stages of ‘fujimino’ grape fruits were acquired from the gene expression omnibus (GEO) database of NCBI (GSE77218) (https://www.ncbi.nlm.nih.gov/geo/query/acc.cgi), which is measured by RNA-sequencing (RNA-Seq) data [76]. During the whole growing season, the fruits of three-year-old ‘fujimino’ grape trees were sampled three times in the green fruit expanding stage (40DAF or DAF40), verison stage (65DAF or DAF65), and ripe stage (90DAF or DAF90). In addition, according to the RNA sequence data downloaded from NCBI geographic data set (accession numbers GSE62744 and GSE62745), the expression of *VvBBX* gene in four berry development stages of 10 different grape varieties were analyzed [44]. The 10 varieties include five red-skinned berries (Sangiovese, Barbera, Negro amaro, Refosco, and Primitivo) and five white-skinned berries (Vermentino, Garganega, Glera, Moscato Bianco, and Passerina). Berries were sampled in three replicates at four developmental stages, the pea-sized berry stage at 20d after flowering, the berries beginning to touch stage just prior to veraison (Pre_veraison), the berry-softening stage at the end of veraison (End_veraison), and the fully ripe berry stage at harvest.

### Plant growth condition and different hormone treatment

The 4-years-old ‘Fujiminori’ grapevine trees grown in the standard field conditions at the Qingdao Agricultural University fruit farm, Qingdao, China, were selected as experimental material. In order to study gene expression characteristics of the *VvBBX* genes during berry development and ripening, grapevine berry samples were also collected at three-time points during the growing season: the green fruit expanding stage (50 DAF), veraison (70 DAF) and ripe/harvest stages (90 DAF).

Ethylene and ABA treatments (500 mg/L ethephon (ETH, an ethylene-releasing reagent) and 100 mg/L ABA in 0.02% (v/v) Tween 20 and 1% (v/v) ethanol (both used as surfactants) were sprayed on grapevine berries at green fruit expanding stage (40 DAF) and at the same time the control berries were sprayed in 0.02% (v/v) Tween 20 and 1% (v/v) ethanol. The ethephon, ABA and control solutions were applied with a handheld sprayer until run-off. During the growing season the berries samples were collected at three-time points: the green fruit expanding stage (50 DAF), veraison (70 DAF) and ripe/harvest stages (90 DAF) from treated and control plants. All samples were collected three times form each sample and were immediately frozen in liquid nitrogen and stored at − 80 °C until used. In addition, in order to study the expression profiles of *VvBBXs* genes*,* in response to GA3 and CPPU, the grapevine RNA-seq data sets were retrieved from the published supplementary data sets [45].

### The expression of *VvBBX* genes under stress condition

In order to study the expression profiles of *VvBBXs* in response to different stress treatment (Cu, salt, waterlogging and drought stress), the grapevine RNA-seq data in response to waterlogging and drought stress were retrieved from NCBI database (SRA accession no. SRP070475 and SRP074162, respectively) (https://www.ncbi.nlm.nih.gov/sra/SRP070475 and https://www.ncbi.nlm.nih.gov/sra/?term=SRP074162) [77, 78]. RNA-seq data for expression profiles in response to Cu and salt were retrieved from published supplemental data sets [37, 79]. Summer black (hybrids of *V. vinifera* and *V. labrusca*) 2-years-old grapevine were used to detect mRNA expression of *VvBBX* under abiotic stresses. Cu stress of potted grapevine plants was simulated with 100 μM CuSO_4_ and salt stress was treated with 0.8% NaCl [37, 79]. Control plants are also treated with distilled water and waterlogging treatment was carried out by immersing the plants in the water for 48 h [78] and drought treatment was carried out by withholding water 20 days [78]. Grapevine plantlets were used as a control and have grown in an appropriate conditions. All types of samples were copied three times, and the expanded leaves from the third and fourth pieces of the shooting vertices were collected from treatment and control group during deep sequencing. The analysis of RNA-seq data was based on the previous method [37], and the RPKM (Reads Per Kilobase per Million mapped reads) values were used to estimate the gene expression level. The heatmap of VvBBX genes was revealed using R software (http://www.bioconductor.org/).

### qRT-PCR analysis

Total RNA samples were extracted by TIANGEN RNAprep pure (Tiangen, Beijing, China) agreeing to the manufacturer’s instructions, and to remove the residual contaminated genomic DNA the RNA was processed with DNase I (RNase free; TaKaRa Biotechnology, Dalian, China). Subsequently, the first-strand cDNA was reversed from 1.5 μg total RNA using PrimeScript RTase (TaKaRa Biotechnology, Dalian, China). The primers that were used for qRT-PCR were designed by Primer 3.0 online software and the primer sequences details were presented in Additional file 17: Table S8. The qRT-PCR was performed using SYBR® Premixm Ex Taq™ (TaKaRa, Japan) with the Applied Biosystems 7500 Real-Time PCR System. All the experiments were carried out with three biological replicates. The relative levels of gene expression were determined by the 2^–ΔΔCT^ methods with Actin (AB073011) serving as a housekeeping gene [80].

### Subcellular localization analysis

In order to verify the subcellular localization of the VvBBX, without the stop codon of four full-length open reading frames (ORFs) of the VvBBX genes (VvBBX1, 9, 13 and 22) were amplified from the cDNAs of four-years-old ‘Fujiminori’ grapevine. Under the control of the CaMV35S promoter, the amplification product is cloned into a pCAMBIA1300 vector with green fluorescence protein (GFP). The gene-specific primers were presented in Additional file 17: Table S8. Through electroporation, four VvBBXs recombinant plasmids were transfected into the *Agrobacterium tumefaciens* strain EH105. Transient detection of *Agrobacterium*-mediated infected into the epidermal cells of *Nicotiana benthamiana*, as described by Sparkes et al. (2006) [81]. The transient expression of VvBBX-GFP was observed using a laser confocal microscopes (Zeiss LSM700, Germany) and the nucleus was visualized using the mCherry-labeled nuclear marker (NF-YA4-m Cherry).

## Supplementary information

**Additional file 1 Figure S1**. Structure of the VvBBX proteins. Numbers indicate amino acid length and position of the corresponding conserved domains. The blue, red and green boxes indicate the B-Box 1, B-Box 2 and CCT domain, respectively. The scale bar represents 100 amino acids.

**Additional file 2 Figure S2**. The conserved domains in the VvBBX proteins. A, B and C represent the protein alignment of the B-Box 1, B-Box 2 and CCT domain, respectively. The x-axis indicates the conserved sequences of the domain. The height of each letter indicates the conservation of each residue across all proteins. The y-axis is a scale of the relative entropy, which reflects the conservation rate of each amino acid.

**Additional file 3 Figure S3**. Multiple sequence alignments of the conserved domains of the VvBBXs. Multiple sequence alignments of the B-box 1 (A), B-box 2 (B) and CCT (C) domains are shown. The sequences were aligned using DNAMAN7.0.

**Additional file 4 Figure S4**. Phylogenetic analysis of BBX genes in grapevine and pear. The full-length amino acid sequences of BBX proteins from grapevine (VvBBX) and pear (PbBBX) were aligned by ClustalX, and the phylogenetic tree was constructed using the maximum likelihood method by MEGA5.0.

**Additional file 5 Figure S5.** Phylogenetic analysis of BBX genes in grapevine, pear, Arabidopsis and tomato. The full-length amino acid sequences of BBX proteins from grapevine (VvBBX), pear (PbBBX), Arabidopsis (AtBBX) and tomato (SlBBX) were aligned by ClustalX, and the phylogenetic tree was constructed using the maximum likelihood method by MEGA5.0.

**Additional file 6 Table S1**. The Ka/Ks ratios and divergence between paralogous VvBBX gene pairs.

**Additional file 7 Figure S6**. Gene structure of the VvBBX family generated from GSDS. The yellow block means the coding sequence (CDS), the blue block means the upstream or downstream of the genes, and the black line indicates the intron. The scale bar indicates the length of the DNA sequences.

**Additional file 8 Figure S7**. Promoter Cis-regulatory elements analysis of grapevine VvBBX genes. Number of each cis-acting element in the promoter region (2.0 kb upstream of the translation start site) of VvBBX genes. Based on the functional annotation, the cis-acting elements were classified into three major classes: plant growth and development, phytohormone responsive, or abiotic and biotic stresses-related cis-acting elements (detailed results shown in Additional file 6: Table S1).

**Additional file 9 Table S2.** Promoter analysis of the grapevine BBX gene family.

**Additional file 10 Table S3.** Expression profiles of the grapevine VvBBX genes in different organs, tissues and developmental stages.

**Additional file 11 Table S4**. Expression profiles of the grapevine VvBBX genes during three fruit developmental stages.

**Additional file 12 Table S5**. Expression profiles of the *VvBBX* genes in response to GA3 and CPPU treatment.

**Additional file 13 Table S6.** Expression profiles of the VvBBX genes in response to abiotic stress.

**Additional file 14 Figure S8.** BBX family members of grapevine, Arabidopsis, rice, tomato and pear.

**Additional file 15 Figure S9.** Protein interaction network of grapevine VvBBX18 and VvBBX19 by STRING.

**Additional file 16 Table S7.** Protein interaction network of grapevine VvBBX18 and VvBBX19 by STRING.

**Additional file 17 Table S8.** The primers sequences of VvBBX genes for qRT-PCR and gene amplification.

## Data Availability

The Arabidopsis, pear and grapevine BBX protein sequences were downloaded from the Arabidopsis information source (TAIR) database (http://www.arabidopsis.org) and GigaDB database (http://gigadb.org/site/index) and Grape Genome CRIBI website (http://genomes.cribi.unipd.it/), respectively. Microarray datas for expression profiles of *V. vinifera* cv ‘Corvina’ (clone48) in different organs at different developmental stages were downloaded from NCBI gene expression omnibus (GEO) datasets (accession numbers: GSE36128) (https://www.ncbi.nlm.nih.gov/geo/query/acc.cgi?acc=GSE36128). RNA-seq data for expression profiles of ‘Fujimino’ grape fruits in developmental stages were acquired from NCBI gene expression omnibus (GEO) database (GSE77218) (https://www.ncbi.nlm.nih.gov/geo/query/acc.cgi). RNA-seq data for expression profiles of 10 different grape varieties in four berry development stages were downloaded from NCBI gene expression omnibus (GEO) database (GSE62744 and GSE62745) (https://www.ncbi.nlm.nih.gov/geo/query/acc.cgi). RNA-seq data in response to GA3 and CPPU were retrieved from the published supplementary data sets (10.1016/j.scienta.2019.02.048). The grapevine RNA-seq data in response to waterlogging and drought stress were retrieved from NCBI database (SRA accession numbers: SRP070475 and SRP074162, respectively) (https://www.ncbi.nlm.nih.gov/sra/SRP070475 and https://www.ncbi.nlm.nih.gov/sra/?term=SRP074162).

## Abbreviations

ABRE: Abscisic acid responsive element
BBX: B-BOX
COP1: Constitutively Photomorphogenic 1
DAF: Days after flowering
ERE: Ethylene-responsive element
FT: Flowering Locus T
GFP: Green fluorescence protein
HMM: Hidden Markov Model
HY5: Protein long hypocotyl 5
MEME: Multiple Em for Motif Elicitation
NJ: Neighbor-joining
ORFs: Open reading frames
PAP1: The production of anthocyanin pigment 1
qRT-PCR: Real-Time PCR
RPKM: Reads Per Kilobase per Million mapped reads
STH2: SALT TOLERANCE HOMOLOG 2
STO: Salt tolerance protein
