# Supplementary figures and images for: Genome-wide identification and analysis of B-BOX gene family in grapevine reveal its potential functions in berry development
Source: BMC Plant Biol. 2020 Feb 13;20:72. doi: 10.1186/s12870-020-2239-3 (PMC7020368; doi:10.1186/s12870-020-2239-3)

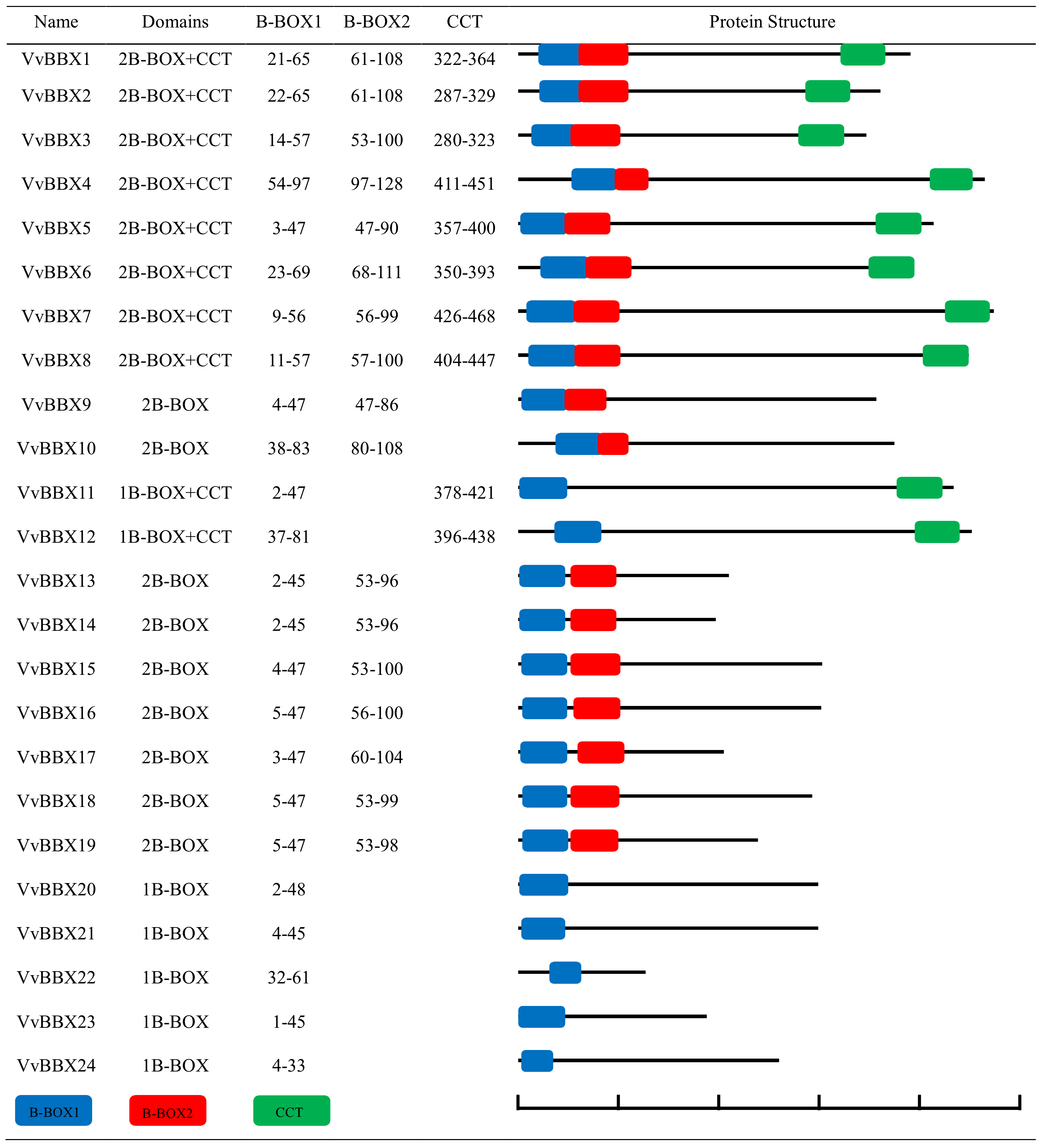


Figure S1

Supplement: Supplementary file 1 — Additional file 1 Figure S1. Structure of the VvBBX proteins. Numbers indicate amino acid length and position of the corresponding conserved domains. The blue, red and green boxes indicate the B-Box 1, B-Box 2 and CCT domain, respectively. The scale bar represents 100 amino acids. [file 12870_2020_2239_MOESM1_ESM.docx]

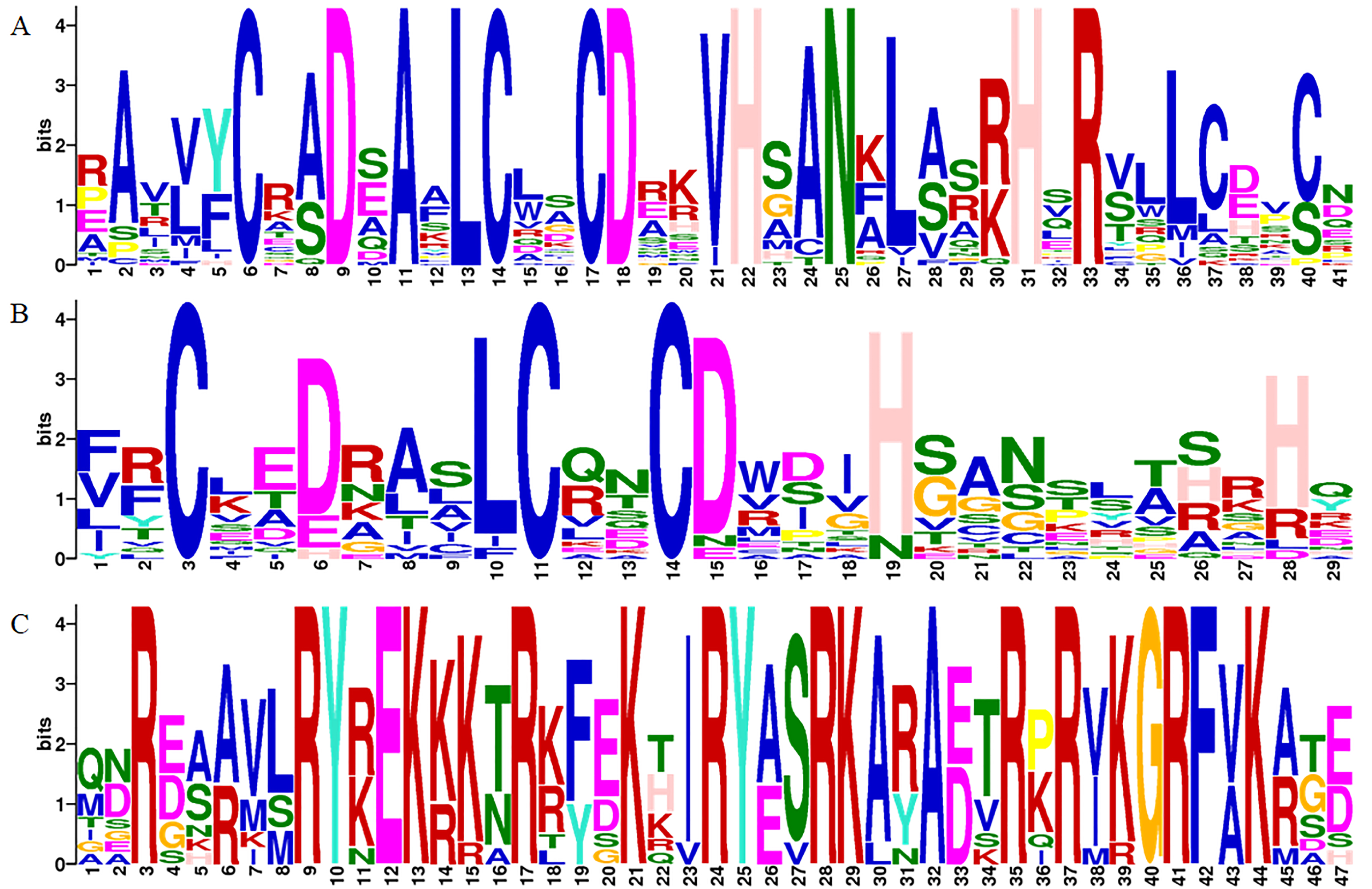


Figure S2

Supplement: Supplementary file 2 — Additional file 2 Figure S2. The conserved domains in the VvBBX proteins. A, B and C represent the protein alignment of the B-Box 1, B-Box 2 and CCT domain, respectively. The x-axis indicates the conserved sequences of the domain. The height of each letter indicates the conservation of each residue across all proteins. The y-axis is a scale of the relative entropy, which reflects the conservation rate of each amino acid. [file 12870_2020_2239_MOESM2_ESM.docx]

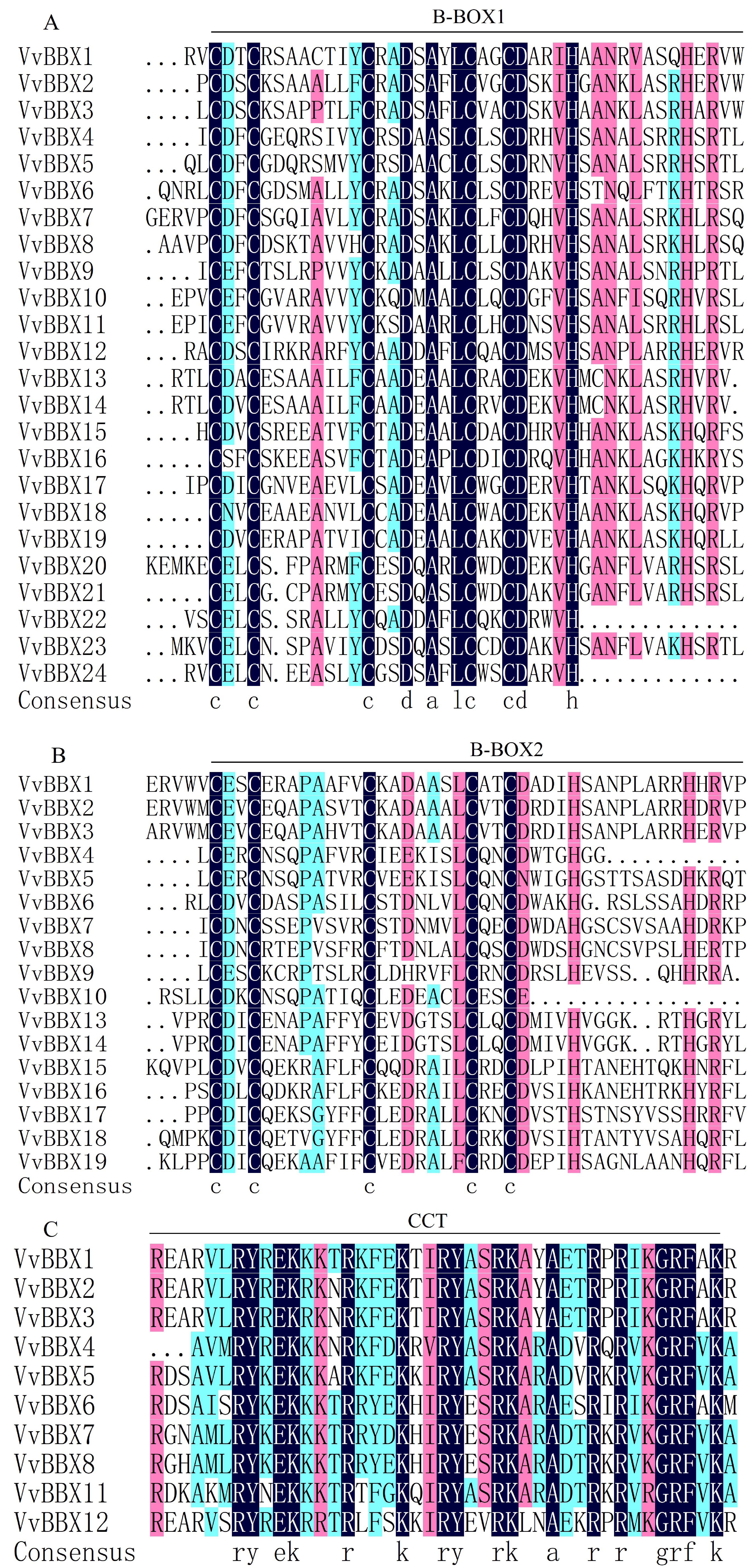


Figure S3

Supplement: Supplementary file 3 — Additional file 3 Figure S3. Multiple sequence alignments of the conserved domains of the VvBBXs. Multiple sequence alignments of the B-box 1 (A), B-box 2 (B) and CCT (C) domains are shown. The sequences were aligned using DNAMAN7.0. [file 12870_2020_2239_MOESM3_ESM.docx]

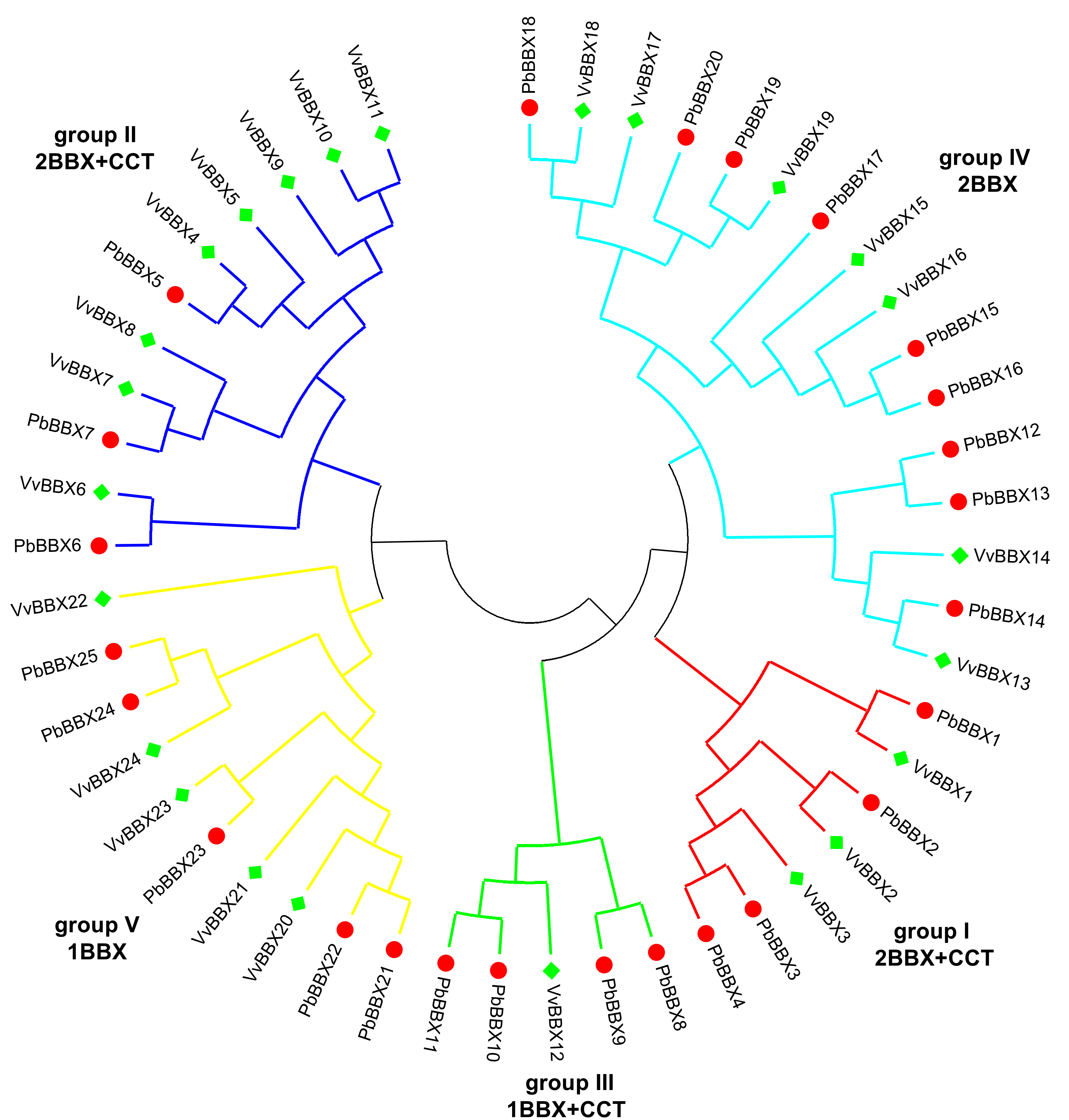


Figure S4

Supplement: Supplementary file 4 — Additional file 4 Figure S4. Phylogenetic analysis of BBX genes in grapevine and pear. The full-length amino acid sequences of BBX proteins from grapevine (VvBBX) and pear (PbBBX) were aligned by ClustalX, and the phylogenetic tree was constructed using the maximum likelihood method by MEGA5.0. [file 12870_2020_2239_MOESM4_ESM.docx]

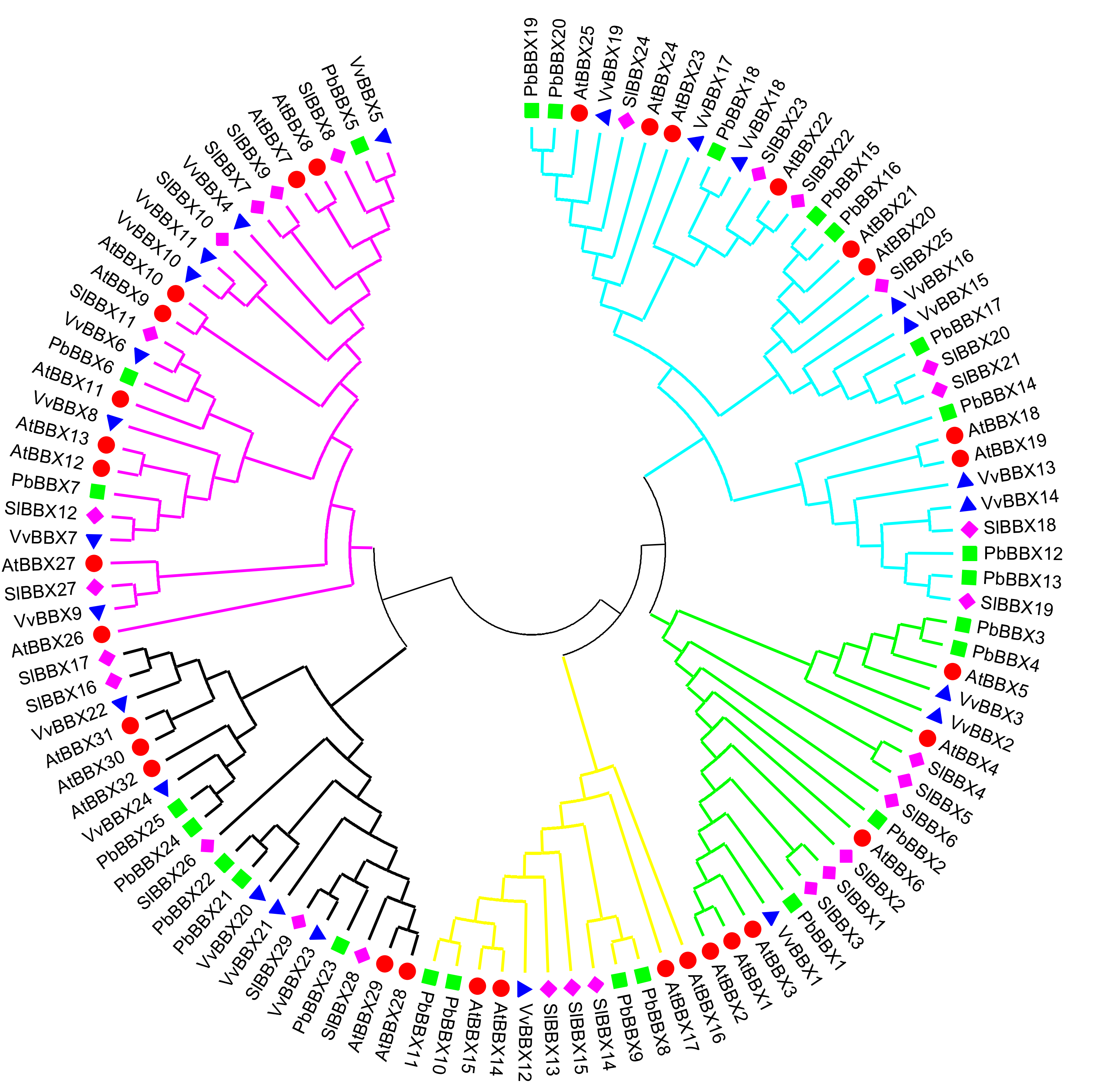


Figure S5

Supplement: Supplementary file 5 — Additional file 5 Figure S5. Phylogenetic analysis of BBX genes in grapevine, pear, Arabidopsis and tomato. The full-length amino acid sequences of BBX proteins from grapevine (VvBBX), pear (PbBBX), Arabidopsis (AtBBX) and tomato (SlBBX) were aligned by ClustalX, and the phylogenetic tree was constructed using the maximum likelihood method by MEGA5.0. [file 12870_2020_2239_MOESM5_ESM.docx]

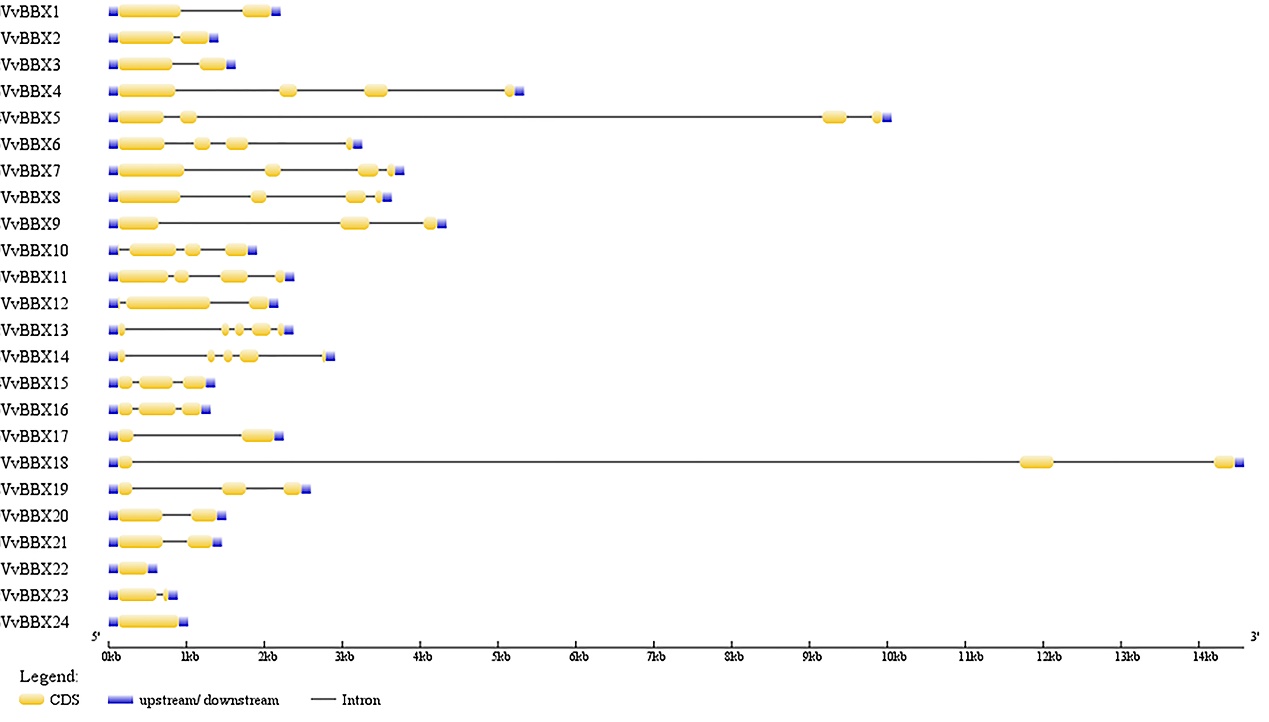


Figure S6

Supplement: Supplementary file 7 — Additional file 7 Figure S6. Gene structure of the VvBBX family generated from GSDS. The yellow block means the coding sequence (CDS), the blue block means the upstream or downstream of the genes, and the black line indicates the intron. The scale bar indicates the length of the DNA sequences. [file 12870_2020_2239_MOESM7_ESM.docx]

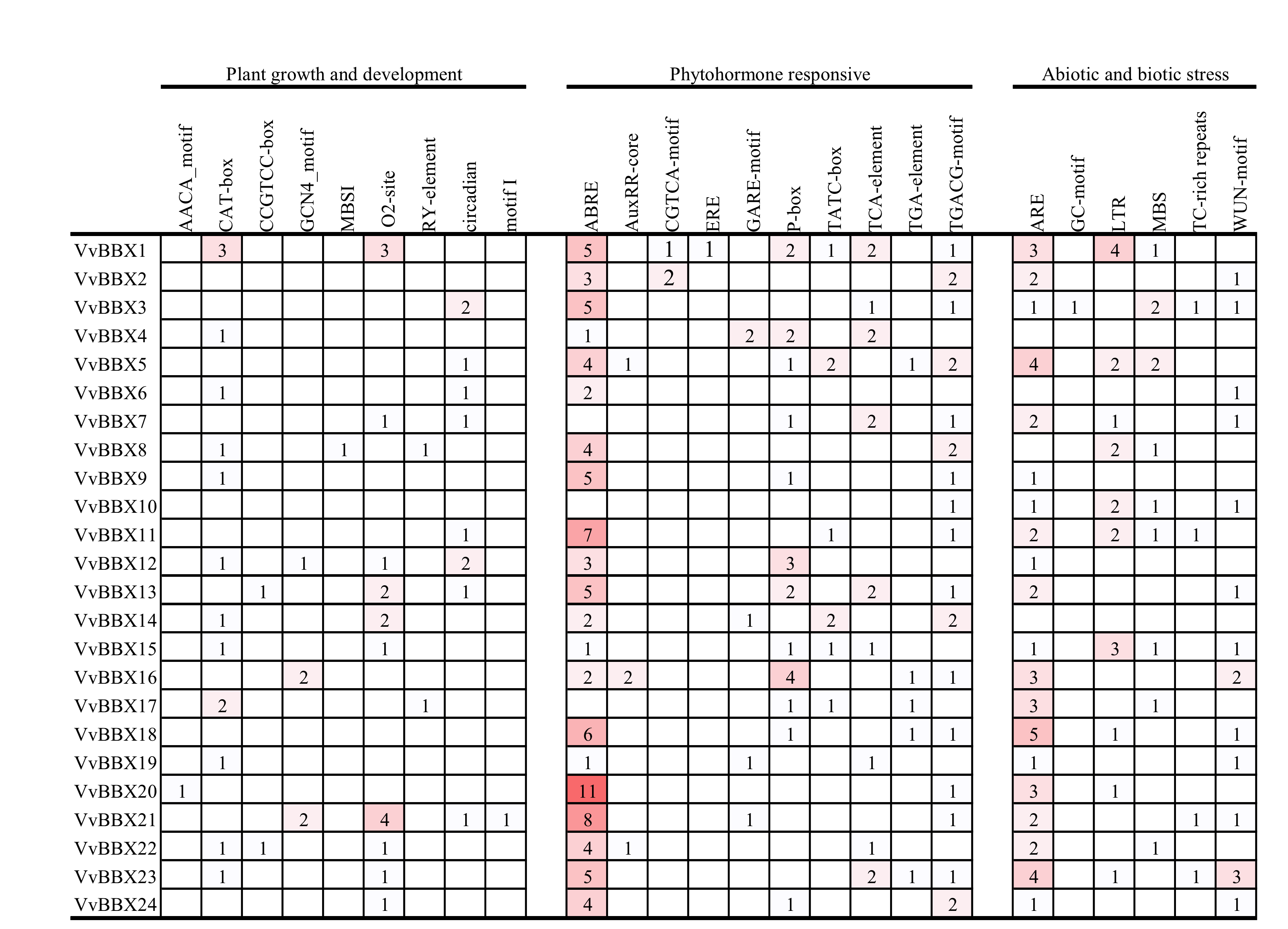


Figure S7

Supplement: Supplementary file 8 — Additional file 8 Figure S7. Promoter Cis-regulatory elements analysis of grapevine VvBBX genes. Number of each cis-acting element in the promoter region (2.0 kb upstream of the translation start site) of VvBBX genes. Based on the functional annotation, the cis-acting elements were classified into three major classes: plant growth and development, phytohormone responsive, or abiotic and biotic stresses-related cis-acting elements (detailed results shown in Additional file 6: Table S1). [file 12870_2020_2239_MOESM8_ESM.docx]

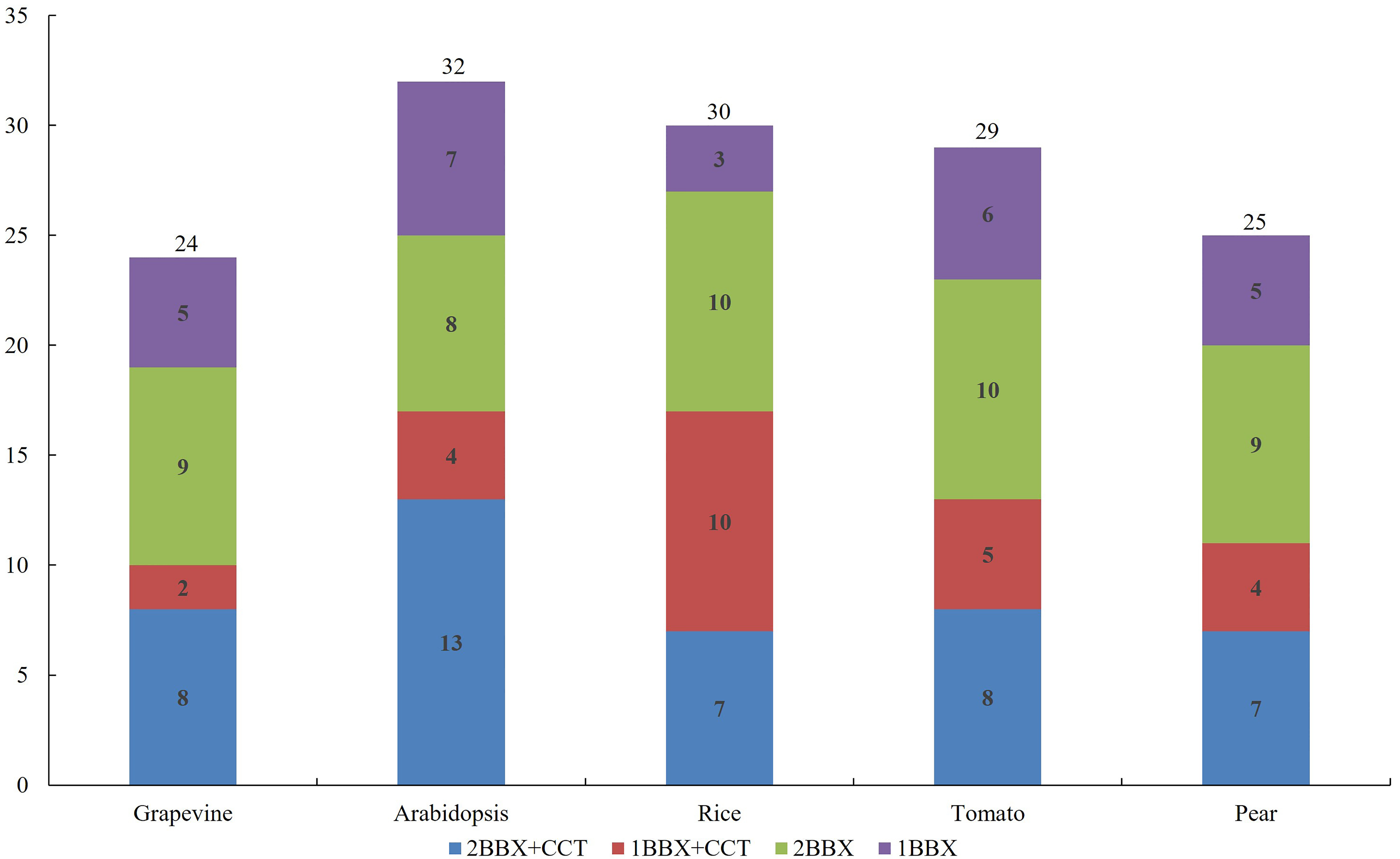


Figure S8

Supplement: Supplementary file 14 — Additional file 14 Figure S8. BBX family members of grapevine, Arabidopsis, rice, tomato and pear. [file 12870_2020_2239_MOESM14_ESM.docx]

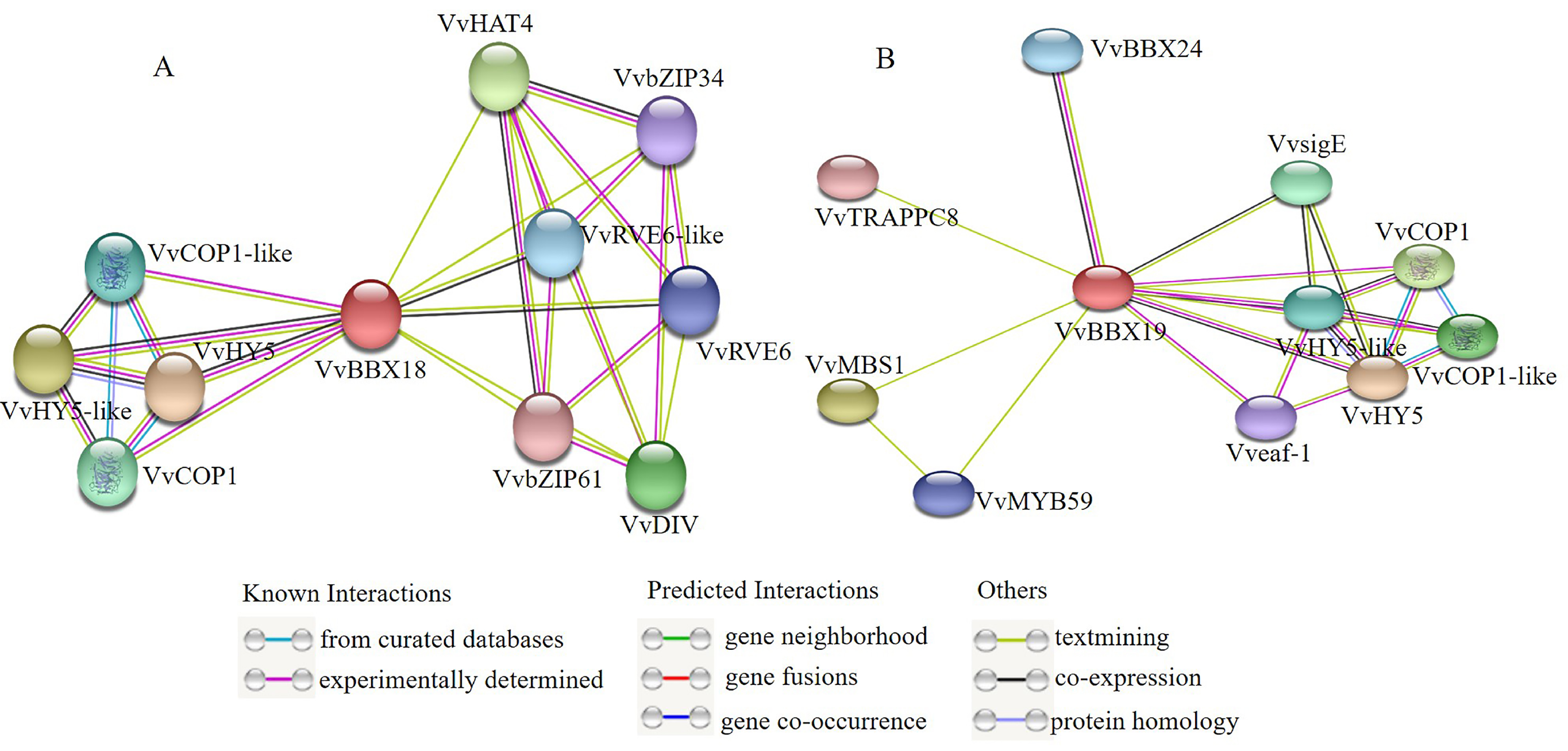


Figure S9

Supplement: Supplementary file 15 — Additional file 15 Figure S9. Protein interaction network of grapevine VvBBX18 and VvBBX19 by STRING. [file 12870_2020_2239_MOESM15_ESM.docx]
