# Supplementary material for: Genome-wide identification and analysis of B-BOX gene family in grapevine reveal its potential functions in berry development
Source: BMC Plant Biol. 2020 Feb 13;20:72. doi: 10.1186/s12870-020-2239-3 (PMC7020368; doi:10.1186/s12870-020-2239-3)
Supplement: Supplementary file 6 — Additional file 6 Table S1. The Ka/Ks ratios and divergence between paralogous VvBBX gene pairs. [file 12870_2020_2239_MOESM6_ESM.docx]

Table S1. The Ka/Ks ratios and divergence between paralogous VvBBX gene pairs.

| Paralogous pairs | Ka | Ks | Ka/Ks |
| --- | --- | --- | --- |
| VvBBX7-VvBBX8 | 0.340815 | 1.91395 | 0.178069 |
| Vv BBX10-VvBBX11 | 0.739295 | 3.6411 | 0.203042 |
| Vv BBX13-VvBBX14 | 0.16804 | 0.920066 | 0.182639 |
| Vv BBX14-VvBBX15 | 0.909911 | 4.03768 | 0.225355 |
| VvBBX15- VvBBX16 | 0.346144 | 2.28615 | 0.151409 |
